# Supplementary material for: Relative burden of lung and pleural cancers from exposure to asbestos: a cross-sectional analysis of occupational mortality in England and Wales
Source: BMJ Open. 2020 Apr 8;10(4):e036319. doi: 10.1136/bmjopen-2019-036319 (PMC7245407; doi:10.1136/bmjopen-2019-036319)
Supplement: Supplementary data [file bmjopen-2019-036319supp001.pdf]

Supplementary Table 1: Job groups with known hazard of COPD, silica or asbestos excluded for weighting analysis

	Job group
**Excluded jobs with known hazard of COPD**	Managers in Transport, Mining and Energy Industries<br>Glass and ceramic workers combined<br>Coal miners combined<br>Moulders, Core Makers, Die Casters<br>Electroplaters combined<br>Other metal manufacturers combined
**Excluded jobs with known hazard from silica**	Chemical workers combined<br>Glass and ceramic workers combined<br>Coal miners combined<br>Moulders, Core Makers, Die Casters<br>Other metal manufacturers combined<br>Bricklayers, Masons combined<br>Mine (excluding coal) & Quarry Workers
**Excluded jobs with known hazard from asbestos**	Vocational Trainers, Social Scientists etc.<br>Chemical Engineers and Scientists<br>Other Professional Engineers<br>Draughtspersons<br>Laboratory Technicians<br>Other Technicians<br>Production and maintenance managers<br>Managers in Construction<br>Fire Service Personnel<br>Chemical workers combined<br>Upholsterers<br>Carpenters & Joiners<br>Cabinet makers combined<br>Smiths & Forge Workers<br>Metal working machine operatives combined<br>Production fitters<br>Electricians electrical maintenance fitters combined<br>Electrical engineers (not professional) combined<br>Plumbers, Heating & Ventilating Engineers & Related Trades<br>Sheet Metal Workers<br>Metal Plate Workers, Shipwrights, Riveters<br>Steel Erectors<br>Scaffolders, Riggers combined<br>Welding Trades<br>Coach and vehicle body builders and repairers combined<br>Other construction workers combined<br>Dockers goods porters and slingers combined<br>Electrical, Energy, Boiler Operatives & Attendants combined
