# Supplementary material for: Relative burden of lung and pleural cancers from exposure to asbestos: a cross-sectional analysis of occupational mortality in England and Wales
Source: BMJ Open. 2020 Apr 8;10(4):e036319. doi: 10.1136/bmjopen-2019-036319 (PMC7245407; doi:10.1136/bmjopen-2019-036319)
Supplement: Supplementary data [file bmjopen-2019-036319supp002.pdf]

**Supplementary Table 2: Asbestos exposed job groups with significantly elevated PMRs for cancer of the pleura over the period 1979-2010**

| Job group | Deaths from all causes 1979-2010 | Cancer of the pleura | | | Lung cancer | | | |
|---|---|---|---|---|---|---|---|---|
| | | Deaths observed | Deaths expected | PMR (95%CI) | Deaths observed | Deaths expected | Deaths expected adjusted for smoking | Adjusted PMR (95%CI) |
| Chemical Engineers and Scientists | 7,111 | 34 | 17.3 | 196 (136-274) | 521 | 525.5 | 525.0 | 99 (91-108) |
| Other Professional Engineers | 48,783 | 174 | 120.3 | 145 (124-168) | 4,307 | 3,614.3 | 3,961.8 | 109 (105-112) |
| Draughtspersons | 14,498 | 45 | 23.4 | 193 (141-258) | 1,062 | 1,324.7 | 1,179.9 | 90 (85-96) |
| Other Technicians | 17,177 | 64 | 31.6 | 203 (156-259) | 1,499 | 1,480.2 | 1,504.3 | 100 (95-105) |
| Production and maintenance managers | 63,504 | 153 | 113.3 | 135 (114-158) | 6,418 | 5,827.7 | 5,490.1 | 117 (114-120) |
| Managers in Construction | 18,079 | 87 | 32.7 | 266 (213-328) | 2,030 | 1,687.6 | 1,589.8 | 128 (122-133) |
| Upholsterers | 5,459 | 32 | 14.4 | 223 (152-314) | 632 | 650.8 | 590.8 | 107 (99-116) |
| Carpenters & Joiners | 68,780 | 582 | 195 | 298 (275-324) | 7,566 | 7,987.3 | 7,387.8 | 102 (100-105) |
| Cabinet makers combined | 9,070 | 35 | 24.1 | 145 (101-202) | 997 | 1,071.8 | 973.0 | 102 (96-109) |
| Metal working machine operatives combined | 112,777 | 315 | 228.3 | 138 (123-154) | 13,450 | 13,588.2 | 12,258.1 | 110 (108-112) |
| Production fitters | 111,536 | 509 | 334.2 | 152 (139-166) | 13,010 | 13,349.8 | 12,347.8 | 105 (104-107) |
| Electricians electrical maintenance fitters combined | 60,353 | 428 | 186.9 | 229 (208-252) | 6,135 | 6,933.1 | 6,175.3 | 99 (97-102) |
| Electrical engineers (not professional) combined | 19,392 | 78 | 49.9 | 156 (123-195) | 1,885 | 2,112.4 | 1,748.9 | 108 (103-113) |
| Plumbers, Heating & Ventilating Engineers & Related Trades | 44,862 | 424 | 136.9 | 310 (281-341) | 5,416 | 5,212.5 | 4,999.7 | 108 (105-111) |
| Sheet Metal Workers | 15,254 | 80 | 42.1 | 190 (151-237) | 1,963 | 1,835.4 | 1,781.4 | 110 (105-115) |
| Metal Plate Workers, Shipwrights, Riveters | 11,831 | 151 | 33.8 | 446 (378-524) | 1,693 | 1,475.7 | 1,449.2 | 117 (111-123) |
| Scaffolders, Riggers combined | 9,703 | 38 | 21.4 | 178 (126-244) | 1,303 | 1,069.9 | 1,099.5 | 119 (112-125) |
| Welding Trades | 30,337 | 142 | 85.0 | 167 (141-197) | 3,897 | 3,555.2 | 3,633.3 | 107 (104-111) |
| Coach and vehicle body builders and repairers combined | 6,452 | 58 | 19.1 | 303 (230-392) | 733 | 739.0 | 696.2 | 105 (98-113) |
| Other construction workers combined | 152,986 | 454 | 356 | 128 (116-140) | 18,810 | 17,186.4 | 17,661.9 | 107 (105-108) |
| Dockers goods porters and slingers combined | 26,426 | 85 | 47.6 | 179 (143-221) | 3,667 | 3,332.1 | 3,215.1 | 114 (110-118) |
| Electrical, Energy, Boiler Operatives & Attendants combined | 15,639 | 70 | 30.3 | 231 (180-292) | 2,113 | 1,989.2 | 1,998.8 | 106 (101-110) |
