# Supplementary material for: Relative burden of lung and pleural cancers from exposure to asbestos: a cross-sectional analysis of occupational mortality in England and Wales
Source: BMJ Open. 2020 Apr 8;10(4):e036319. doi: 10.1136/bmjopen-2019-036319 (PMC7245407; doi:10.1136/bmjopen-2019-036319)
Supplement: Supplementary data [file bmjopen-2019-036319supp003.pdf]

Supplementary Table 3: Ratio of excess deaths from lung cancer to observed deaths from cancer of the pleura, by job group and time period

	Ratio of excess deaths from lung cancer to observed deaths from cancer of the pleura (95%CI)			
**Job group**	**Whole period 1979-2010**	**Period 1 1979-1990**	**Period 2 1991-2000**	**Period 3 2001-2010**
Chemical Engineers and Scientists	-0.12 (-1.52,1.30)	1.76 (-0.43,5.41)	-0.31 (-2.41,1.81)	-3.04 (-11.57,0.27)
Other Professional Engineers	1.98 (1.18,2.84)	3.57 (1.97,5.69)	1.11 (0.10,2.22)	1.46 (-0.13,3.29)
Draughtspersons	-2.62 (-4.58,-1.16)	-4.14 (-9.12,-1.62)	-0.64 (-3.40,1.68)	-3.05 (-8.36,0.06)
Other Technicians	-0.08 (-1.34,1.18)	2.32 (-3.04,4.68)	0.01 (-1.23,1.24)	-1.28 (-3.71,0.74)
Production and maintenance managers	6.06 (4.79,7.6)	6.76 (4.68,9.77)	5.28 (3.38,8.14)	5.31 (3.06,8.82)
Managers in Construction	5.06 (3.76,6.82)	5.79 (3.55,9.67)	4.62 (2.59,8.43)	4.70 (2.89,8.01)
Upholsterers	1.29 (-0.28,3.17)	1.84 (-0.44,5.17)	-0.62 (-3.47,2.31)	2.83 (-0.86,12.12)
Carpenters & Joiners	0.31 (0,0.60)	0.98 (0.29,1.72)	-0.14 (-0.63,0.32)	0.16 (-0.27,0.58)
Cabinet makers combined	0.69 (-1.14,2.59)	-3.92 (-15.09,1.43)	0.04 (-2.42,2.63)	4.21 (1.50,10.07)
Metal working machine operatives combined	3.78 (2.99,4.73)	4.53 (3.06,6.48)	3.93 (2.68,5.53)	2.32 (1.13,3.87)
Production fitters	1.30 (0.83,1.78)	1.48 (0.62,2.41)	0.77 (0.14,1.37)	1.58 (0.62,2.61)
Electricians electrical maintenance fitters combined	-0.09 (-0.47,0.26)	-0.16 (-0.89,0.56)	0.23 (-0.48,0.92)	-0.17 (-0.69,0.32)
Electrical engineers (not professional) combined	1.74 (0.60,3.10)	0.57 (-1.27,2.69)	0.76 (-1.06,2.72)	5.53 (2.95,11.86)
Plumbers, Heating & Ventilating Engineers & Related Trades	0.98 (0.63,1.35)	2.15 (1.41,3.00)	0.53 (-0.01,1.07)	0.37 (-0.21,0.96)
Sheet Metal Workers	2.27 (1.16,3.52)	3.64 (0.94,7.57)	2.58 (0.85,5.40)	0.69 (-0.61,2.30)
Metal Plate Workers, Shipwrights, Riveters	1.61 (1.03,2.31)	1.96 (1.11,2.99)	1.27 (0.25,2.60)	1.17 (0.23,2.40)
Scaffolders, Riggers combined	5.36 (3.24,8.80)	2.83 (-0.65,9.13)	8.55 (4.75,17.67)	4.25 (1.09,10.43)
Welding Trades	1.86 (0.95,2.85)	1.26 (-0.20,2.84)	1.14 (-0.29,2.81)	3.71 (1.95,6.59)
Coach and vehicle body builders and repairers combined	0.63 (-0.25,1.62)	1.41 (0.03,3.28)	0.64 (-0.93,2.46)	-1.08 (-4.00,1.21)
Other construction workers combined	2.53 (1.91,3.16)	4.72 (3.43,6.15)	2.87 (1.90,4.03)	-0.02 (-1.09,1.04)
Dockers goods porters and slingers combined	5.32 (3.62,7.48)	7.06 (4.22,11.43)	3.79 (1.93,6.61)	4.13 (0.03,11.73)
Electrical, Energy, Boiler Operatives & Attendants combined	1.63 (0.29,3.16)	2.71 (0.75,5.29)	0.11 (-2.16,2.72)	1.30 (-1.37,5.04)
