# Supplementary material for: Relative burden of lung and pleural cancers from exposure to asbestos: a cross-sectional analysis of occupational mortality in England and Wales
Source: BMJ Open. 2020 Apr 8;10(4):e036319. doi: 10.1136/bmjopen-2019-036319 (PMC7245407; doi:10.1136/bmjopen-2019-036319)
Supplement: Author's manuscript [file bmjopen-2019-036319.draft_revisions.pdf]

BMJ Open

BMJ Open is committed to open peer review. As part of this commitment we make the peer review history of every article we publish publicly available.

When an article is published we post the peer reviewers' comments and the authors' responses online. We also post the versions of the paper that were used during peer review. These are the versions that the peer review comments apply to.

The versions of the paper that follow are the versions that were submitted during the peer review process. They are not the versions of record or the final published versions. They should not be cited or distributed as the published version of this manuscript.

BMJ Open is an open access journal and the full, final, typeset and author-corrected version of record of the manuscript is available on our site with no access controls, subscription charges or pay-per-view fees (http://bmjopen.bmj.com).

If you have any questions on BMJ Open's open peer review process please email info.bmjopen@bmj.com

BMJ Open

Relative burden of lung and pleural cancers from occupational exposure to asbestos

Journal:	*BMJ Open*
Manuscript ID	bmjopen-2019-036319
Article Type:	Original research
Date Submitted by the Author:	10-Dec-2019
Complete List of Authors:	Harris, Elizabeth; University of Southampton, MRC Lifecourse Epidemiology Unit; University of Southampton, MRC Versus Arthritis Centre for Musculoskeletal Health and Work<br>D'Angelo, Stefania; University of Southampton, MRC Lifecourse Epidemiology Unit; University of Southampton, MRC Versus Arthritis Centre for Musculoskeletal Health and Work<br>Darnton, Andrew; Health and Safety Executive Bootle Headquarters, Statistics and Epidemiology Unit, Science Directorate<br>Coggon, David ; University of Southampton, MRC Lifecourse Epidemiology Unit; University of Southampton, MRC Versus Arthritis Centre for Musculoskeletal Health and Work
Keywords:	EPIDEMIOLOGY, OCCUPATIONAL & INDUSTRIAL MEDICINE, Epidemiology < ONCOLOGY, Respiratory tract tumours < ONCOLOGY

SCHOLARONE™ Manuscripts

BMJ

I, the Submitting Author has the right to grant and does grant on behalf of all authors of the Work (as defined in the below author licence), an exclusive licence and/or a non-exclusive licence for contributions from authors who are: i) UK Crown employees; ii) where BMJ has agreed a CC-BY licence shall apply, and/or iii) in accordance with the terms applicable for US Federal Government officers or employees acting as part of their official duties; on a worldwide, perpetual, irrevocable, royalty-free basis to BMJ Publishing Group Ltd ("BMJ") its licensees and where the relevant Journal is co-owned by BMJ to the co-owners of the Journal, to publish the Work in this journal and any other BMJ products and to exploit all rights, as set out in our licence.

The Submitting Author accepts and understands that any supply made under these terms is made by BMJ to the Submitting Author unless you are acting as an employee on behalf of your employer or a postgraduate student of an affiliated institution which is paying any applicable article publishing charge ("APC") for Open Access articles. Where the Submitting Author wishes to make the Work available on an Open Access basis (and intends to pay the relevant APC), the terms of reuse of such Open Access shall be governed by a Creative Commons licence – details of these licences and which Creative Commons licence will apply to this Work are set out in our licence referred to above.

Other than as permitted in any relevant BMJ Author's Self Archiving Policies, I confirm this Work has not been accepted for publication elsewhere, is not being considered for publication elsewhere and does not duplicate material already published. I confirm all authors consent to publication of this Work and authorise the granting of this licence.

For peer review only

Relative burden of lung and pleural cancers from occupational exposure to asbestos

E Clare Harris[1,2]*, Stefania D'Angelo[1,2], Andrew Darnton[3], David Coggon[1,2]

[1]MRC Lifecourse Epidemiology Unit, University of Southampton, Southampton, UK

[2]Arthritis Research UK/MRC Centre for Musculoskeletal Health and Work, University of Southampton, Southampton, UK

[3]Statistics and Epidemiology Unit, Science Directorate, Health and Safety Executive, Bootle, UK

*Corresponding author

Elizabeth Clare Harris

MRC Lifecourse Epidemiology Unit (University of Southampton)

Southampton General Hospital

Tremona Road

Southampton

SO16 6YD

E-mail: ech@mrc.soton.ac.uk

Telephone: 02380 777624

Word count: 2,124

For peer review only

I, the Submitting Author has the right to grant and does grant on behalf of all authors of the Work (as defined in the below author licence), an exclusive licence and/or a non-exclusive licence for contributions from authors who are: i) UK Crown employees; ii) where BMJ has agreed a CC-BY licence shall apply, and/or iii) in accordance with the terms applicable for US Federal Government officers or employees acting as part of their official duties; on a worldwide, perpetual, irrevocable, royalty-free basis to BMJ Publishing Group Ltd ("BMJ") its licensees and where the relevant Journal is co-owned by BMJ to the co-owners of the Journal, to publish the Work in BMJ Open and any other BMJ products and to exploit all rights, as set out in our licence.

The Submitting Author accepts and understands that any supply made under these terms is made by BMJ to the Submitting Author unless you are acting as an employee on behalf of your employer or a postgraduate student of an affiliated institution which is paying any applicable article publishing charge ("APC") for Open Access articles. Where the Submitting Author wishes to make the Work available on an Open Access basis (and intends to pay the relevant APC), the terms of reuse of such Open Access shall be governed by a Creative Commons licence – details of these licences and which Creative Commons licence will apply to this Work are set out in our licence referred to above.

For peer review only

Abstract

Objectives:

To explore whether asbestos-exposed jobs vary importantly in the ratio of excess mortality from lung cancer to deaths from pleural cancer.

Methods:

Using data on underlying cause of death and last full-time occupation for 3,688,916 deaths among men aged 20-74 years in England and Wales during 1979-2010, we calculated proportional mortality ratios (PMRs), standardised for age and social class, with all occupations combined as the reference. For each of 22 asbestos-exposed job groups with significantly elevated PMRs for cancer of the pleura, we calculated excess mortality from lung cancer (observed minus expected deaths) and its ratio to the number of deaths from cancer of the pleura. To reduce confounding effects of smoking, we adjusted the expected deaths from lung cancer in each job group, according to a formula based on its PMR for chronic obstructive pulmonary disease (COPD).

Results:

Adjusted PMRs for lung cancer were elevated in all but four of the 22 asbestos-exposed job-groups, with the overall excess of lung cancer 1.7 times the number of deaths from pleural cancer. However, the ratio of excess lung cancer to deaths from pleural cancer varied widely between job groups, being significantly greater than the overall ratio in six, and significantly less in seven.

Conclusions:

Excess lung cancer in asbestos-exposed jobs is not in simple proportion to deaths from pleural cancer, and the ratio may vary importantly according to the intensity of exposure to different types of asbestos. National burdens of lung cancer from occupational exposure to asbestos may not be so high as previously thought.

Key words: Lung cancer, pleural cancer, asbestos, mortality, occupation

For peer review only

Strengths and limitations of this study

- Use of national data covering more than 30 years gave excellent statistical precision.
- Confounding by differences in smoking habits between occupations was addressed by a novel method of adjustment based on PMRs for COPD.
- By adjusting PMRs for social class, we reduced the potential for bias because overall mortality in a job group was unusually high or low.
- There was potential for bias from misclassification of occupations and causes of death, but misclassification of lung cancer and COPD as causes of death is likely to have been non-differential with respect to occupation, and therefore to have biased PMRs for those diseases towards the null.
- There was incomplete ascertainment of pleural cancers before 2001 because deaths ascribed to mesothelioma without any specified anatomical location (most of which would have been pleural) were classed along with other cancers of unknown origin, but a separate analysis for 2001-10 that included unspecified mesotheliomas supported the main study findings.

For peer review only

Introduction

Estimating the population burden of lung cancer from occupational exposure to asbestos is complicated by uncertainty about the distribution of exposures across occupations and the potential for confounding by smoking. One approach has been to assume that impact is in proportion to the occurrence of mesothelioma. For example, when modelling future numbers of asbestos-related lung tumours in the Netherlands, Van der Bij and colleagues applied a multiplier of 1.5 to deaths from mesothelioma[1] – a factor which they derived from an earlier

meta-analysis of 55 cohort studies of asbestos workers.[2] Implicit in such calculations is an assumption that the ratio of excess lung cancer to mesothelioma does not vary importantly according to intensity and duration of exposure to different types of asbestos, and should therefore be similar across different jobs. To test the validity of that assumption, we estimated and compared such ratios for 22 asbestos-exposed job groups, using data from a national analysis of proportionate mortality by occupation.

Methods

The Office for National Statistics (ONS) provided us with data on underlying cause of death and last full-time occupation for 3,688,916 deaths among men aged 20-74 years in England and Wales during 1979-2010 (excluding 1981 when records were incomplete). From these, we calculated proportional mortality ratios (PMRs), standardised for age (in five-year bands), social class (six categories) and calendar period (1979-90, 1991-2000, 2001-10), for occupational categories (job groups) classified as in earlier analyses,[3] taking all occupations combined as the standard.

To address possible confounding by smoking, the prevalence of which varies by occupation, we used PMRs for chronic obstructive pulmonary disease (COPD) to adjust expected numbers of deaths from lung cancer. We first excluded job groups with excess mortality from one or more of COPD, cancer of the pleura or peritoneum, asbestosis or silicosis, which was likely to have arisen from exposures in those jobs (Supplementary Table 1). For the 106 job groups that remained (which were presumed to have no major occupational hazard of lung cancer or COPD), we confirmed that the PMR for lung cancer was linearly related to that for COPD by calculation of a Spearman correlation coefficient, and then fitted a weighted linear regression model of the form:

$$(\text{PMR for lung cancer}) = a * (\text{PMR for COPD}) + b \qquad \{1\}$$

For peer review only

For this purpose, the weighting was according to the expected number of deaths from COPD in each job group.

Next, we focused on 22 asbestos-exposed job groups with significantly elevated PMRs over the period 1979-2010 for cancer of the pleura (ICD9 163, ICD10 C38.4, C38.8 and C45.0, lower 95% confidence limit > 100) (Supplementary Table 2). For these job groups, we used the regression coefficients, a and b, from {1} to adjust expected numbers of deaths from lung cancer according to the PMR for COPD. Thus, the expected number of deaths was multiplied by {a * (PMR for COPD) + b}.

With this correction, we calculated the excess of lung cancer for each job group (observed – expected deaths), and its ratio to the observed number of deaths from cancer of the pleura. Confidence intervals for ratios were computed through random simulations (1000 per estimate) in which we assumed that the expected number of deaths from lung cancer was constant, while the numbers of deaths from lung cancer and cancer of the pleura each followed a Poisson distribution with mean equal to the observed number of deaths from that cancer in our dataset.

During 1979-2000, when ICD 9 was used to classify causes of death, there was no separate diagnostic category for mesotheliomas with unspecified anatomical origin, and they were included in a much larger grouping of “malignant neoplasms without specification of site”. However, ICD 10, which was used during 2001-10, included unique codes for mesothelioma including C45.9 for “mesothelioma unspecified”. In a sensitivity analysis, we repeated our calculations for this period, aggregating all deaths from mesotheliomas other than of the peritoneum (C45.2, C45.7 and C45.9) with those from pleural cancer.

In addition, PMRs for deaths where mesothelioma was mentioned anywhere in the death certificate text were available for the periods 1980, 1982-2000, and 2002-2010, from national

For peer review only

statistics published by the Health and Safety Executive (HSE).[4] In further sensitivity analyses, we related excess mortality from lung cancer by job group to excess deaths from mesothelioma in these data (adjusting the ratios to account for there being slightly fewer years of data on mesothelioma).

Results

In the 106 job groups with no major hazard of COPD, silicosis or asbestos-related disease, PMRs for lung cancer correlated strongly with those for COPD (Spearman correlation coefficient = 0.78, Figure 1). The weighted regression equation was:

(PMR for lung cancer) = 0.57*(PMR for COPD) + 42.

When the coefficients from this equation were used to adjust expected numbers of lung cancer deaths in the 22 job groups with significantly high PMRs for pleural cancer (Supplementary Table 2), the PMR for lung cancer was elevated in all but four, and the overall excess of lung cancer was 1.69 times the number of deaths from pleural cancer. However, the ratio between excess deaths from lung cancer and deaths from pleural cancer varied between job groups, such that in six it was significantly greater than the overall average, and in seven significantly less (Figure 2).

During 2001-2010, 3061 deaths from mesotheliomas other than of the peritoneum were recorded in the 22 asbestos-exposed job groups of interest, in addition to the 1205 classed as pleural cancer. Inclusion of these additional deaths in our calculations gave a lower overall ratio (0.28), but again indicated substantial heterogeneity between job groups (Figure 3). Moreover the job groups with the highest and lowest ratios were much the same as in the previous analysis.

For peer review only

Similar results were obtained in the analysis based on deaths with any mention of mesothelioma in the death certificate text. The overall ratio (in this case to excess rather than total deaths from mesothelioma) was 1.13 for the full study period, and 0.46 for 2001-2010, with similar variation in the ratios for specific job groups.

Discussion

Our analysis indicates that among occupations entailing exposure to asbestos, the ratio between excess deaths from lung cancer and deaths from pleural cancer/mesothelioma can vary substantially. This suggests that burdens of lung cancer attributable to asbestos are not in simple proportion to numbers of mesotheliomas, and that the ratio may vary importantly according to the pattern of exposures within a population.

We limited our investigation to men since asbestos-related disease was much less frequent among women. Moreover, only 30% of the women who died in the early part of the study period (1979-1990) had occupations recorded on their death certificates.[5]

Our use of national data covering more than 30 years gave excellent statistical precision, but there was potential for bias from misclassification of occupations and causes of death. In the UK, death certificates document only the last full-time occupation, but for chronic diseases with long induction periods (such as lung and pleural cancer), jobs held earlier in life may be more relevant. Furthermore, occupations and causes of death are not always assigned accurately.[6] Nevertheless, we think it unlikely that such errors could account for the variation in ratios of excess lung cancer to pleural cancer that we observed.

The 22 job groups on which we focused in our main analysis were those that we could be reasonably confident were associated with an asbestos hazard. However, it was not essential that they should account for all asbestos-related cancer in the study population. Any under-ascertainment of cases attributable to work in those jobs, either because of migration to other employment or through misclassification of occupations on death certificates, would reduce

For peer review only

both the excess mortality from lung cancer and the number of deaths from pleural cancer. However, it would not be expected to bias the ratio of those measures differentially across job groups.

Misclassification of lung cancer and COPD as causes of death is likely to have been non-differential with respect to occupation, and therefore to have biased PMRs for those diseases towards the null. It is reassuring, however, that after exclusion of job groups with exposure to known causes of lung cancer and/or COPD, we observed a strong correlation between PMRs for the two diseases (r = 0.78). This suggests that such misclassification was not a major problem.

A greater concern was the incomplete ascertainment of mesotheliomas before 2001 in our main dataset. This occurred because at that time, deaths ascribed to mesothelioma without any specified anatomical location (most of which would have been pleural) were classed along with other cancers of unknown origin. Data from 2001-10, when they were assigned to a specific code, indicated that they outnumbered deaths ascribed to pleural cancer more than twofold. Thus, variation in the extent of under-ascertainment by job group could have caused serious bias. However, when we restricted our analysis to 2001-10, and included mesotheliomas other than of the peritoneum with pleural cancers, there was still marked variation in their frequency relative to excess lung cancer. And importantly, the job groups with the highest and lowest ratios were much the same. Moreover, similar heterogeneity was observed in our analysis based on deaths with any mention of mesothelioma on the death certificate.

As with all analyses of proportionate mortality, there was a possibility that expected numbers of deaths from specific causes of death could be biased if overall mortality in a job group were unusually high or low. However, in stratifying our analyses by social class, we reduced the potential for large variation between job groups in total mortality, and it seems unlikely that such bias could explain differences in the ratio of excess lung cancer to pleural cancer of the magnitude that we observed.

For peer review only

A particular challenge in studying occupational mortality from lung cancer is the scope for confounding by differences in smoking habits between occupations. To address that problem, we adjusted expected deaths from lung cancer according to the PMR for COPD in the job group under consideration. In deriving the formula for the adjustment, we took care to exclude job groups with exposure to major occupational causes of either COPD or lung cancer, in the expectation that the variation between job groups in PMRs would then be driven largely by differences in smoking. The strength of the correlation that we found between the two diseases supported that assumption, and although not all cases of COPD are picked up from death certificates (because of competing causes of death), it seems that the PMR from COPD did provide a meaningful proxy for smoking, making our expected numbers of deaths more reliable than would have been the case without adjustment.

We know from other research that smoking and asbestos interact in causing lung cancer, such that relative risks from the two causes approximately multiply.[7] It follows, that in a person with lung cancer who has been both a smoker and exposed to asbestos, the disease may be attributable to both causes (or put another way, avoidance of either of the exposures might have been sufficient to prevent the disease). However, with the method of statistical analysis that we employed, interactions between smoking and asbestos could be ignored. The parameter on which we focused was the difference between the number of deaths from lung cancer that actually occurred in the job group and the number that would have been expected if the job group had the smoking habits that it did, but no exposure to asbestos. That measure will have included excess deaths attributable to asbestos alone in non-smokers, and to the joint effects of smoking and asbestos as compared with smoking alone in smokers.

The variability that we found in the ratio of excess lung cancer to mesothelioma by job group may in part reflect differences by type of asbestos. Previous meta-analysis of cohort studies has suggested a lower ratio for crocidolite (0.7) than for chrysotile (6.1), amosite (4.0) and mixed fibres (1.9).[2] However, intensity and timing of exposure could also be a factor, and might explain why, when we included mesotheliomas other than of the peritoneum, the mean

For peer review only

ratio that we observed across all 22 job groups (0.28) was relatively low. Another analysis, based on national data for England and Wales during 1980-2000, suggested an intermediate ratio in the order of 0.67-1.0.[8] The disparity from our estimate may in part reflect differences in the methods used to control for confounding effects of smoking, but there may also have been changes over time. Together, these two investigations suggest that national burdens of lung cancer from occupational exposure to asbestos may not be so high as previously has been thought.

The potential for variability in the ratio of excess lung cancer to mesothelioma should be taken into account when estimating population burdens of the disease from occupational exposure to asbestos.

Acknowledgements

We thank the staff of the Office for National Statistics, who provided us with the data files for our analysis, and Vanessa Cox, who helped with computer analysis.

Funding statement

This work was supported by Unit core funding from the Medical Research Council grant numbers 10.13039/5011000002 65, MRC_MC_UP_A620_1018, MRC_MC_UU_12011/5 (https://mrc.ukri.org/).

Competing interests

The authors have declared that no competing interests exist.

For peer review only

Authors contributions

DC designed the study, ECH and DC acquired the ONS data, AD the HSE data, and all authors developed the methodology and analysis. SD and AD carried out the analyses, ECH and DC wrote the first draft of the manuscript, and all authors revised and approved the final version. The Office for National Statistics provided us with the data files for our analysis, and Vanessa Cox assisted with computer analysis.

Data sharing

The data underlying the results presented in the study can be made available to other researchers subject to agreement from the Office for National Statistics.

Data relevant to the specific occupations in this analysis are provided in the supporting information file: Supplementary Table 2.

2001-2010 occupational mortality data for England and Wales is available at https://www.ons.gov.uk/peoplepopulationandcommunity/healthandsocialcare/healthinequalities/adhocs/007958occupationalmortalityinenglandandwales2001to2010

1991-2000 occupational mortality data for England and Wales is available at https://webarchive.nationalarchives.gov.uk/20160129235354/http://www.ons.gov.uk/ons/publications/re-reference-tables.html?edition=tcm%3A77-168405

Patient and Public Involvement

This research was done without patient or public involvement.

For peer review only

References

1 Van der Bij S, Vermeulen RCH, Portengen L, et al. Expected number of asbestos-related lung cancers in the Netherlands in the next two decades: a comparison of methods. *Occup Environ Med* 2016;73:342-49.

2 McCormack V, Peto J, Byrnes G, et al. Estimating the asbestos-related lung cancer burden from mesothelioma mortality. *Br J Cancer* 2012;106:575-84.

3 Harris EC, Palmer KT, Cox V, et al. Trends in mortality from occupational hazards among men in England and Wales during 1979-2010. *Occup Environ Med* 2016;73:385-93.

4 Health and Safety Executive. Mesothelioma mortality by occupation: Mesothelioma deaths in Great Britain for 2011-2017 and 2002-2010 by occupation. Available from: http://www.hse.gov.uk/statistics/causdis/mesothelioma/mesothelioma-mortality-by-occupation-2002-2015.pdf Accessed November 2019.

5 Inskip H, Coggon D, Winter P, et al. (1995) Occupational mortality of women. In: Drever F, ed. Occupational Health: Decennial Supplement. London: HMSO 1995:44-61.

6 Alderson MR. Some sources of error in British occupational mortality data. *Br J Indust Med* 1972;29:245-54.

7 Ngamwong Y, Tangamornsuksan W, Lohitnavy O, et al. Additive Synergism between Asbestos and Smoking in Lung Cancer Risk: A Systematic Review and Meta-Analysis. *PLoS One* 2015;10(8):e0135798.

8 Darnton AJ, McElvenny DM, Hodgson JT. Estimating the number of asbestos-related lung cancer deaths in Great Britain from 1980 to 2000. *Ann Occup Hyg* 2006;50:29-38.

Figure legends

For peer review only

Figure 1. PMRs for lung cancer and COPD in job groups with no major occupational exposure to causes of either disease: men in England and Wales aged 20-74 years, 1979-80 and 1982-2010.

Figure footnote: The areas of the circles represent the expected number of deaths from COPD in each job group over the study period. The regression line of PMR for lung cancer against PMR for COPD is from an analysis that weighted according to the expected number of deaths from COPD in each job group over the study period (see text).

Figure 2. Ratios of estimated excess deaths from lung cancer to observed deaths from cancer of pleura, 1979-80 and 1982-2010.

Figure footnote: a) Figures in brackets are observed numbers of deaths/corresponding PMRs for cancer of the pleura. b) Bars represent 95% confidence intervals, and the vertical line indicates the average ratio across all 22 job groups of 1.69.

Figure 3. Ratios of estimated excess deaths from lung cancer to observed deaths from cancer of pleura and mesothelioma, 2001-10.

Figure footnote: a) Figures in brackets are observed numbers of deaths/corresponding PMRs for cancer of the pleura and mesothelioma. b) Bars represent 95% confidence intervals, and the vertical line indicates the average ratio across all 22 job groups of 0.28.

For peer review only

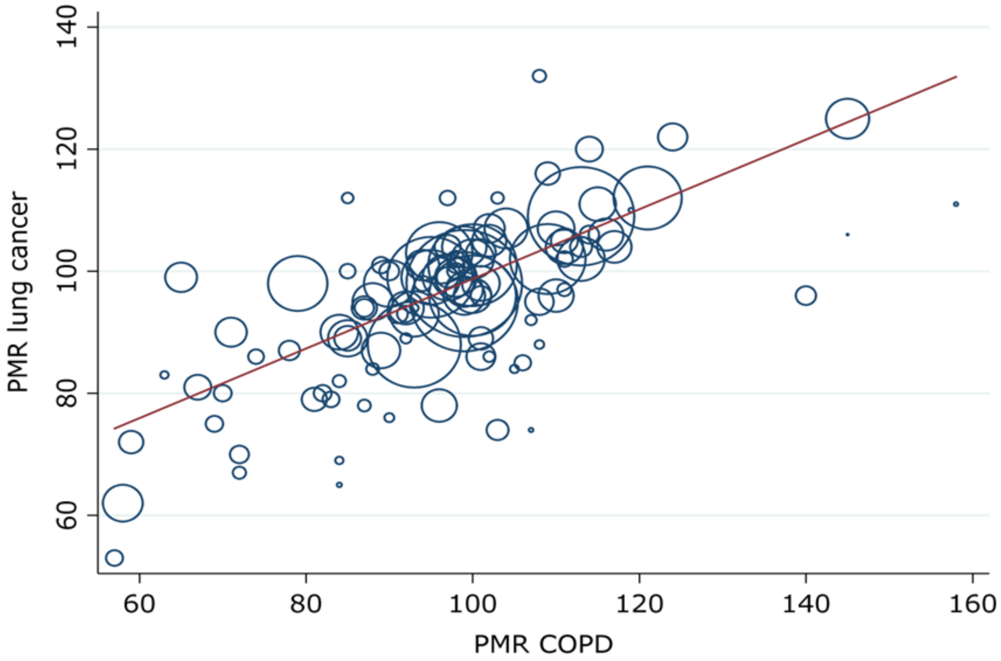

For peer review only

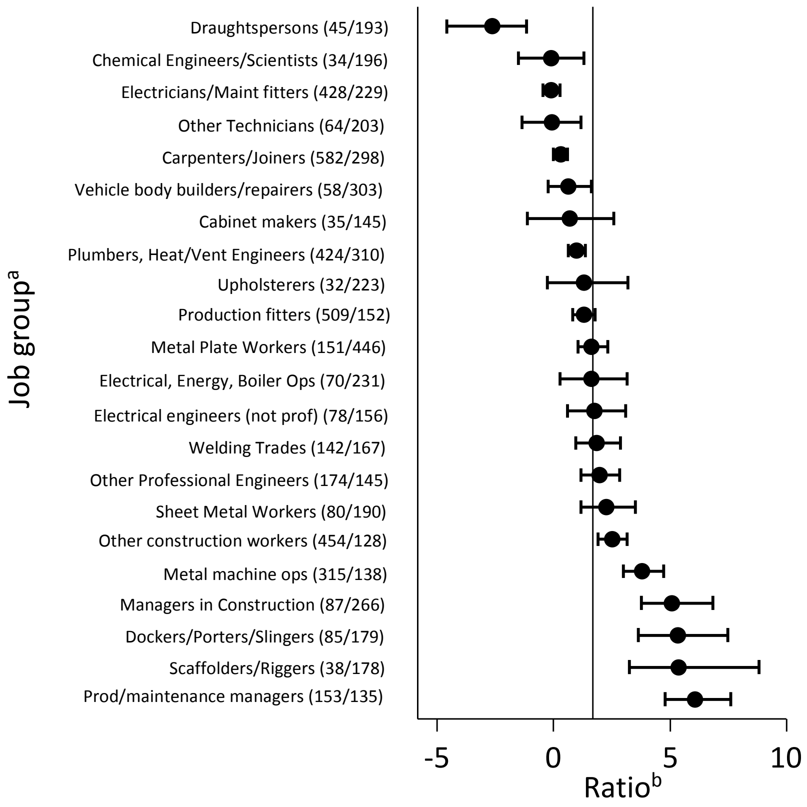
Draughtspersons (45/193)
Chemical Engineers/Scientists (34/196)
Electricians/Maint fitters (428/229)
Other Technicians (64/203)
Carpenters/Joiners (582/298)
Vehicle body builders/repairers (58/303)
Cabinet makers (35/145)
Plumbers, Heat/Vent Engineers (424/310)
Upholsterers (32/223)
Production fitters (509/152)
Metal Plate Workers (151/446)
Electrical, Energy, Boiler Ops (70/231)
Electrical engineers (not prof) (78/156)
Welding Trades (142/167)
Other Professional Engineers (174/145)
Sheet Metal Workers (80/190)
Other construction workers (454/128)
Metal machine ops (315/138)
Managers in Construction (87/266)
Dockers/Porters/Slingers (85/179)
Scaffolders/Riggers (38/178)
Prod/maintenance managers (153/135)
Job group[a]
-5
0
5
10
Ratio[b]

review only

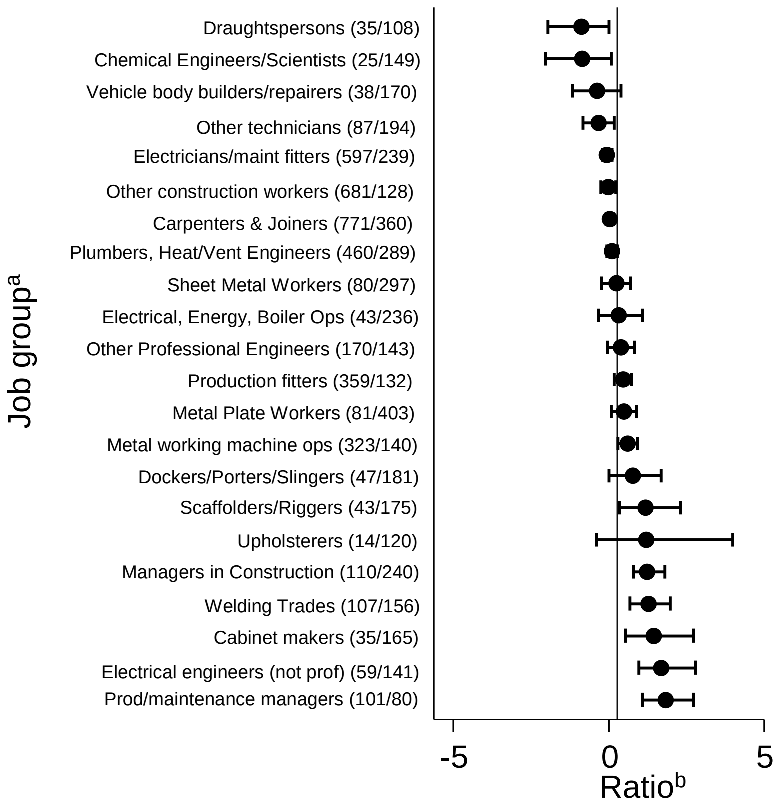
Job group[a]
Draughtspersons (35/108)
Chemical Engineers/Scientists (25/149)
Vehicle body builders/repairers (38/170)
Other technicians (87/194)
Electricians/maint fitters (597/239)
Other construction workers (681/128)
Carpenters & Joiners (771/360)
Plumbers, Heat/Vent Engineers (460/289)
Sheet Metal Workers (80/297)
Electrical, Energy, Boiler Ops (43/236)
Other Professional Engineers (170/143)
Production fitters (359/132)
Metal Plate Workers (81/403)
Metal working machine ops (323/140)
Dockers/Porters/Slingers (47/181)
Scaffolders/Riggers (43/175)
Upholsterers (14/120)
Managers in Construction (110/240)
Welding Trades (107/156)
Cabinet makers (35/165)
Electrical engineers (not prof) (59/141)
Prod/maintenance managers (101/80)
-5
0
5
Ratio[b]

review only

Supplementary Table 1: Job groups with known hazard of COPD, silica or asbestos excluded for weighting analysis

	Job group
**Excluded jobs with known hazard of COPD**	Managers in Transport, Mining and Energy Industries<br>Glass and ceramic workers combined<br>Coal miners combined<br>Moulders, Core Makers, Die Casters<br>Electroplaters combined<br>Other metal manufacturers combined
**Excluded jobs with known hazard from silica**	Chemical workers combined<br>Glass and ceramic workers combined<br>Coal miners combined<br>Moulders, Core Makers, Die Casters<br>Other metal manufacturers combined<br>Bricklayers, Masons combined<br>Mine (excluding coal) & Quarry Workers
**Excluded jobs with known hazard from asbestos**	Vocational Trainers, Social Scientists etc.<br>Chemical Engineers and Scientists<br>Other Professional Engineers<br>Draughtspersons<br>Laboratory Technicians<br>Other Technicians<br>Production and maintenance managers<br>Managers in Construction<br>Fire Service Personnel<br>Chemical workers combined<br>Upholsterers<br>Carpenters & Joiners<br>Cabinet makers combined<br>Smiths & Forge Workers<br>Metal working machine operatives combined<br>Production fitters<br>Electricians electrical maintenance fitters combined<br>Electrical engineers (not professional) combined<br>Plumbers, Heating & Ventilating Engineers & Related Trades<br>Sheet Metal Workers<br>Metal Plate Workers, Shipwrights, Riveters<br>Steel Erectors<br>Scaffolders, Riggers combined<br>Welding Trades<br>Coach and vehicle body builders and repairers combined<br>Other construction workers combined<br>Dockers goods porters and slingers combined<br>Electrical, Energy, Boiler Operatives & Attendants combined

For peer review only

Supplementary Table 2: Asbestos exposed job groups with significantly elevated PMRs for cancer of the pleura over the period 1979-2010

Job group	Deaths from all causes 1979-2010	Cancer of the pleura			Lung cancer			
		Deaths observed	Deaths expected	PMR (95%CI)	Deaths observed	Deaths expected	Deaths expected adjusted for smoking	Adjusted PMR (95%CI)
Chemical Engineers and Scientists	7,111	34	17.3	196 (136-274)	521	525.5	525.0	99 (91-108)
Other Professional Engineers	48,783	174	120.3	145 (124-168)	4,307	3,614.3	3,961.8	109 (105-112)
Draughtspersons	14,498	45	23.4	193 (141-258)	1,062	1,324.7	1,179.9	90 (85-96)
Other Technicians	17,177	64	31.6	203 (156-259)	1,499	1,480.2	1,504.3	100 (95-105)
Production and maintenance managers	63,504	153	113.3	135 (114-158)	6,418	5,827.7	5,490.1	117 (114-120)
Managers in Construction	18,079	87	32.7	266 (213-328)	2,030	1,687.6	1,589.8	128 (122-133)
Upholsterers	5,459	32	14.4	223 (152-314)	632	650.8	590.8	107 (99-116)
Carpenters & Joiners	68,780	582	195	298 (275-324)	7,566	7,987.3	7,387.8	102 (100-105)
Cabinet makers combined	9,070	35	24.1	145 (101-202)	997	1,071.8	973.0	102 (96-109)
Metal working machine operatives combined	112,777	315	228.3	138 (123-154)	13,450	13,588.2	12,258.1	110 (108-112)
Production fitters	111,536	509	334.2	152 (139-166)	13,010	13,349.8	12,347.8	105 (104-107)
Electricians electrical maintenance fitters combined	60,353	428	186.9	229 (208-252)	6,135	6,933.1	6,175.3	99 (97-102)
Electrical engineers (not professional) combined	19,392	78	49.9	156 (123-195)	1,885	2,112.4	1,748.9	108 (103-113)
Plumbers, Heating & Ventilating Engineers & Related Trades	44,862	424	136.9	310 (281-341)	5,416	5,212.5	4,999.7	108 (105-111)
Sheet Metal Workers	15,254	80	42.1	190 (151-237)	1,963	1,835.4	1,781.4	110 (105-115)
Metal Plate Workers, Shipwrights, Riveters	11,831	151	33.8	446 (378-524)	1,693	1,475.7	1,449.2	117 (111-123)
Scaffolders, Riggers combined	9,703	38	21.4	178 (126-244)	1,303	1,069.9	1,099.5	119 (112-125)
Welding Trades	30,337	142	85.0	167 (141-197)	3,897	3,555.2	3,633.3	107 (104-111)
Coach and vehicle body builders and repairers combined	6,452	58	19.1	303 (230-392)	733	739.0	696.2	105 (98-113)
Other construction workers combined	152,986	454	356	128 (116-140)	18,810	17,186.4	17,661.9	107 (105-108)
Dockers goods porters and slingers combined	26,426	85	47.6	179 (143-221)	3,667	3,332.1	3,215.1	114 (110-118)
Electrical, Energy, Boiler Operatives & Attendants combined	15,639	70	30.3	231 (180-292)	2,113	1,989.2	1,998.8	106 (101-110)

For peer review only

BMJ Open

Relative burden of lung and pleural cancers from exposure to asbestos: a cross-sectional analysis of occupational mortality in England and Wales

Journal:	*BMJ Open*
Manuscript ID	bmjopen-2019-036319.R1
Article Type:	Original research
Date Submitted by the Author:	14-Feb-2020

Complete List of Authors:	Harris, Elizabeth; University of Southampton, MRC Lifecourse Epidemiology Unit; University of Southampton, MRC Versus Arthritis Centre for Musculoskeletal Health and Work<br>D'Angelo, Stefania; University of Southampton, MRC Lifecourse Epidemiology Unit; University of Southampton, MRC Versus Arthritis Centre for Musculoskeletal Health and Work<br>Darnton, Andrew; Health and Safety Executive Bootle Headquarters, Statistics and Epidemiology Unit, Science Directorate<br>Coggon, David ; University of Southampton, MRC Lifecourse Epidemiology Unit; University of Southampton, MRC Versus Arthritis Centre for Musculoskeletal Health and Work

**Primary Subject Heading**:	Epidemiology
Secondary Subject Heading:	Occupational and environmental medicine
Keywords:	EPIDEMIOLOGY, OCCUPATIONAL & INDUSTRIAL MEDICINE, Epidemiology < ONCOLOGY, Respiratory tract tumours < ONCOLOGY

SCHOLARONE™
Manuscripts

I, the Submitting Author has the right to grant and does grant on behalf of all authors of the Work (as defined in the below author licence), an exclusive licence and/or a non-exclusive licence for contributions from authors who are: i) UK Crown employees; ii) where BMJ has agreed a CC-BY licence shall apply, and/or iii) in accordance with the terms applicable for US Federal Government officers or employees acting as part of their official duties; on a worldwide, perpetual, irrevocable, royalty-free basis to BMJ Publishing Group Ltd ("BMJ") its licensees and where the relevant Journal is co-owned by BMJ to the co-owners of the Journal, to publish the Work in this journal and any other BMJ products and to exploit all rights, as set out in our licence.

The Submitting Author accepts and understands that any supply made under these terms is made by BMJ to the Submitting Author unless you are acting as an employee on behalf of your employer or a postgraduate student of an affiliated institution which is paying any applicable article publishing charge ("APC") for Open Access articles. Where the Submitting Author wishes to make the Work available on an Open Access basis (and intends to pay the relevant APC), the terms of reuse of such Open Access shall be governed by a Creative Commons licence – details of these licences and which Creative Commons licence will apply to this Work are set out in our licence referred to above.

Other than as permitted in any relevant BMJ Author's Self Archiving Policies, I confirm this Work has not been accepted for publication elsewhere, is not being considered for publication elsewhere and does not duplicate material already published. I confirm all authors consent to publication of this Work and authorise the granting of this licence.

For peer review only

Relative burden of lung and pleural cancers from exposure to asbestos: a cross-sectional analysis of occupational mortality in England and Wales

E Clare Harris[1,2]*, Stefania D'Angelo[1,2], Andrew Darnton[3], David Coggon[1,2]

[1]MRC Lifecourse Epidemiology Unit, University of Southampton, Southampton, UK

[2]MRC Versus Arthritis Centre for Musculoskeletal Health and Work, University of Southampton, Southampton, UK

[3]Statistics and Epidemiology Unit, Science Directorate, Health and Safety Executive, Bootle, UK

*Corresponding author

Elizabeth Clare Harris

MRC Lifecourse Epidemiology Unit (University of Southampton)

Southampton General Hospital

Tremona Road

Southampton

SO16 6YD

E-mail: ech@mrc.soton.ac.uk

Telephone: 02380 777624

Word count: 2,337

For peer review only

The Submitting Author has the right to grant and does grant on behalf of all authors of the Work (as defined in the below author licence), an exclusive licence and/or a non-exclusive licence for contributions from authors who are: i) UK Crown employees; ii) where BMJ has agreed a CC-BY licence shall apply, and/or iii) in accordance with the terms applicable for US Federal Government officers or employees acting as part of their official duties; on a worldwide, perpetual, irrevocable, royalty-free basis to BMJ Publishing Group Ltd ("BMJ") its licensees and where the relevant Journal is co-owned by BMJ to the co-owners of the Journal, to publish the Work in BMJ Open and any other BMJ products and to exploit all rights, as set out in our licence.

The Submitting Author accepts and understands that any supply made under these terms is made by BMJ to the Submitting Author unless you are acting as an employee on behalf of your employer or a postgraduate student of an affiliated institution which is paying any applicable article publishing charge ("APC") for Open Access articles. Where the Submitting Author wishes to make the Work available on an Open Access basis (and intends to pay the relevant APC), the terms of reuse of such Open Access shall be governed by a Creative Commons licence – details of these licences and which Creative Commons licence will apply to this Work are set out in our licence referred to above.

For peer review only

Abstract

Objectives:

To explore the extent to which asbestos-exposed jobs vary in the ratio of excess mortality from lung cancer to deaths from pleural cancer.

Design:

Using data on underlying cause of death and last full-time occupation for 3,688,916 deaths among men aged 20-74 years in England and Wales during 1979-2010, we calculated proportional mortality ratios (PMRs), standardised for age and social class, with all occupations combined as reference. For each of 22 asbestos-exposed job groups with significantly elevated PMRs for pleural cancer, we calculated excess mortality from lung cancer (observed minus expected deaths) and its ratio to number of deaths from pleural cancer. To reduce confounding effects of smoking, we adjusted expected deaths from lung cancer in each job group, according to a formula based on its PMR for chronic obstructive pulmonary disease (COPD).

Setting:

England and Wales

Participants:

3,688,916 men who died aged 20-74 years during 1979-2010

Outcome measures:

Ratios of excess mortality from lung cancer to deaths from pleural cancer by job group

Results:

Adjusted PMRs for lung cancer were elevated in all but four of the 22 asbestos-exposed job-groups, but the ratio of excess lung cancer to deaths from pleural cancer varied widely between job groups, being significantly greater than the overall ratio in six, and significantly less in seven. Analysis for 2001-2010, when (because of changes in coding) ascertainment of pleural tumours was more reliable, showed similar variation between job groups, and indicated an overall ratio of 0.28.

Conclusions:

For peer review only

Excess lung cancer in asbestos-exposed jobs is not in simple proportion to deaths from pleural cancer, and the ratio may vary importantly according to intensity of exposure to different types of asbestos and concomitant smoking habits. The current burden of lung cancer from occupational exposure to asbestos in Britain may not be so high as previously thought.

Key words: Lung cancer, pleural cancer, asbestos, mortality, occupation

For peer review only

Strengths and limitations of this study

- Use of national data covering more than 30 years gave excellent statistical precision.
- Confounding by differences in smoking habits between occupations was addressed by a novel method of adjustment based on PMRs for COPD.
- By adjusting PMRs for social class, we reduced the potential for bias because overall mortality in a job group was unusually high or low.
- There was potential for bias from misclassification of occupations and causes of death, but misclassification of lung cancer and COPD as causes of death is likely to have been non-differential with respect to occupation, and therefore to have biased PMRs for those diseases towards the null.
- There was incomplete ascertainment of pleural cancers before 2001 because deaths ascribed to mesothelioma without any specified anatomical location (most of which would have been pleural) were classed along with other cancers of unknown origin, but a separate analysis for 2001-10 that included unspecified mesotheliomas supported the main study findings.

For peer review only

Introduction

Quantifying the population burden of lung cancer from occupational exposure to asbestos is important for prioritisation of control measures, planning future healthcare provision, and assessing the impact of preventive strategies. Attributable numbers of deaths have been estimated in several countries including Great Britain,[1,2] Italy,[3] and Argentina, Brazil, Colombia and Mexico.[4] However, the task is complicated by uncertainty about the distribution of exposures across occupations, and the potential for confounding by smoking.

One approach has been to assume that impact is in proportion to the occurrence of mesothelioma. For example, when modelling future numbers of asbestos-related lung tumours in the Netherlands, Van der Bij and colleagues applied a multiplier of 1.5 to deaths from mesothelioma[5] – a factor which they derived from an earlier meta-analysis of 55 cohort studies of asbestos workers.[6] Others have suggested somewhat lower ratios of 0.55,[7] between 0.67 and 1,[1] and 1.1.[3]

One reason for variation in the ratio could be differences in smoking habits, both between countries and within a single country over time, since the combined effects of asbestos and smoking on risk of lung cancer appear to be more than additive.[8,9] In addition, the ratio of excess lung cancer to mesothelioma may vary according to intensity and duration of exposure to different types of asbestos.[6] If so, such variation could lead to differences between asbestos-exposed occupations, according to the nature of their asbestos exposure.

To explore how much the ratio of excess mortality from lung cancer to deaths from mesothelioma differs between occupations, we estimated and compared such ratios for 22 asbestos-exposed job groups, using data from a national analysis of proportionate mortality by occupation in England and Wales. As part of the analysis, we applied a novel method to adjust for potential confounding effects of smoking.

Methods

The Office for National Statistics (ONS) provided us with data on underlying cause of death and last full-time occupation for 3,688,916 deaths among men aged 20-74 years in England and Wales during 1979-2010 (excluding 1981 when records were incomplete). From these, we calculated proportional mortality ratios (PMRs), standardised for age (in five-year bands), social class (six categories) and calendar period (1979-90, 1991-2000, 2001-10), for

For peer review only

occupational categories (job groups) classified as in earlier analyses,[10] taking all occupations combined as the standard.

To address possible confounding by smoking, the prevalence of which varies by occupation, we used PMRs for chronic obstructive pulmonary disease (COPD) to adjust expected numbers of deaths from lung cancer. We first excluded job groups with excess mortality from one or more of COPD, cancer of the pleura or peritoneum, asbestosis or silicosis, which was likely to have arisen from exposures in those jobs (Supplementary Table 1). For the 106 job groups that remained (which were presumed to have no major occupational hazard of lung cancer or COPD), we confirmed that the PMR for lung cancer was linearly related to that for COPD by calculation of a Spearman correlation coefficient, and then fitted a weighted linear regression model of the form:

$$\text{(PMR for lung cancer)} = a * \text{(PMR for COPD)} + b \qquad \{1\}$$

For this purpose, the weighting was according to the expected number of deaths from COPD in each job group.

Next, we focused on 22 asbestos-exposed job groups with significantly elevated PMRs over the period 1979-2010 for cancer of the pleura (ICD9 163, ICD10 C38.4, C38.8 and C45.0, lower 95% confidence limit > 100) (Supplementary Table 2). For these job groups, we used the regression coefficients, a and b, from {1} to adjust expected numbers of deaths from lung cancer according to the PMR for COPD. Thus, the expected number of deaths was multiplied by {a * (PMR for COPD) + b}.

With this correction, we calculated the excess of lung cancer for each job group (observed – expected deaths), and its ratio to the observed number of deaths from cancer of the pleura. Confidence intervals for ratios were computed through random simulations (1000 per estimate) in which we assumed that the expected number of deaths from lung cancer was constant, while the numbers of deaths from lung cancer and cancer of the pleura each

For peer review only - http://bmjopen.bmj.com/site/about/guidelines.xhtml

followed a Poisson distribution with mean equal to the observed number of deaths from that cancer in our dataset.

During 1979-2000, when ICD 9 was used to classify causes of death, there was no separate diagnostic category for mesotheliomas with unspecified anatomical origin, and they were included in a much larger grouping of “malignant neoplasms without specification of site”. However, ICD 10, which was used during 2001-10, included unique codes for mesothelioma including C45.9 for “mesothelioma unspecified”. In a sensitivity analysis, we repeated our calculations for this period, aggregating all deaths from mesotheliomas other than of the peritoneum (C45.2, C45.7 and C45.9) with those from pleural cancer.

In addition, PMRs for deaths where mesothelioma was mentioned anywhere in the death certificate text were available for the periods 1980, 1982-2000, and 2002-2010, from national statistics published by the Health and Safety Executive (HSE).[11] In further sensitivity analyses, we related excess mortality from lung cancer by job group to excess deaths from mesothelioma in these data (adjusting the ratios to account for there being slightly fewer years of data on mesothelioma).

Patient and Public Involvement

This research was done without patient or public involvement.

Results

In the 106 job groups with no major hazard of COPD, silicosis or asbestos-related disease, PMRs for lung cancer correlated strongly with those for COPD (Spearman correlation coefficient = 0.78, Figure 1). The weighted regression equation was:

(PMR for lung cancer) = 0.57*(PMR for COPD) + 42.

For peer review only

When the coefficients from this equation were used to adjust expected numbers of lung cancer deaths in the 22 job groups with significantly high PMRs for pleural cancer (Supplementary Table 2), the PMR for lung cancer was elevated in all but four, and the overall excess of lung cancer was 1.69 times the number of deaths from pleural cancer. However, the ratio between excess deaths from lung cancer and deaths from pleural cancer varied between job groups, such that in six it was significantly greater than the overall average, and in seven significantly less (Figure 2).

During 2001-2010, 3061 deaths from mesotheliomas other than of the peritoneum were recorded in the 22 asbestos-exposed job groups of interest, in addition to the 1205 classed as pleural cancer. Inclusion of these additional deaths in our calculations gave a lower overall ratio (0.28), but again indicated substantial heterogeneity between job groups (Figure 3). Moreover the job groups with the highest and lowest ratios were much the same as in the previous analysis.

Similar results were obtained in the analysis based on deaths with any mention of mesothelioma in the death certificate text. The overall ratio (in this case to excess rather than total deaths from mesothelioma) was 1.13 for the full study period, and 0.46 for 2001-2010, with similar variation in the ratios for specific job groups.

Discussion

Our analysis indicates that among occupations entailing exposure to asbestos, the ratio between excess deaths from lung cancer and deaths from pleural cancer/mesothelioma can vary substantially. This suggests that burdens of lung cancer attributable to asbestos are not in simple proportion to numbers of mesotheliomas, and that the ratio may vary importantly according to the pattern of exposures within a population, and perhaps also smoking habits.

For peer review only

We limited our investigation to men since asbestos-related disease was much less frequent among women. Moreover, only 30% of the women who died in the early part of the study period (1979-1990) had occupations recorded on their death certificates.[12]

Our use of national data covering more than 30 years gave excellent statistical precision, but there was potential for bias from misclassification of occupations and causes of death. In the UK, death certificates document only the last full-time occupation, but for chronic diseases with long induction periods (such as lung and pleural cancer), jobs held earlier in life may be more relevant. Furthermore, occupations and causes of death are not always assigned accurately.[13] Nevertheless, we think it unlikely that such errors could account for the variation in ratios of excess lung cancer to pleural cancer that we observed.

The 22 job groups on which we focused in our main analysis were those that we could be reasonably confident were associated with an asbestos hazard. However, it was not essential that they should account for all asbestos-related cancer in the study population. Any under-ascertainment of cases attributable to work in those jobs, either because of migration to other employment or through misclassification of occupations on death certificates, would reduce both the excess mortality from lung cancer and the number of deaths from pleural cancer. However, it would not be expected to bias the ratio of those measures differentially across job groups.

Misclassification of lung cancer and COPD as causes of death is likely to have been non-differential with respect to occupation, and therefore to have biased PMRs for those diseases towards the null. It is reassuring, however, that after exclusion of job groups with exposure to

For peer review only

known causes of lung cancer and/or COPD, we observed a strong correlation between PMRs for the two diseases (r = 0.78). This suggests that such misclassification was not a major problem.

A greater concern was the incomplete ascertainment of mesotheliomas before 2001 in our main dataset. This occurred because at that time, deaths ascribed to mesothelioma without any specified anatomical location (most of which would have been pleural) were classed along with other cancers of unknown origin. Data from 2001-10, when they were assigned to a specific code, indicated that they outnumbered deaths ascribed to pleural cancer more than twofold. Thus, variation in the extent of under-ascertainment by job group could have caused serious bias. However, when we restricted our analysis to 2001-10, and included mesotheliomas other than of the peritoneum with pleural cancers, there was still marked variation in their frequency relative to excess lung cancer. And importantly, the job groups with the highest and lowest ratios were much the same. Moreover, similar heterogeneity was observed in our analysis based on deaths with any mention of mesothelioma on the death certificate.

As with all analyses of proportionate mortality, there was a possibility that expected numbers of deaths from specific causes of death could be biased if overall mortality in a job group were unusually high or low. However, in stratifying our analyses by social class, we reduced the potential for large variation between job groups in total mortality, and it seems unlikely that such bias could explain differences in the ratio of excess lung cancer to pleural cancer of the magnitude that we observed.

A particular challenge in studying occupational mortality from lung cancer is the scope for confounding by differences in smoking habits between occupations. To address that problem,

For peer review only - http://bmjopen.bmj.com/site/about/guidelines.xhtml

we adjusted expected deaths from lung cancer according to the PMR for COPD in the job group under consideration. In deriving the formula for the adjustment, we took care to exclude job groups with exposure to major occupational causes of either COPD or lung cancer, in the expectation that the variation between job groups in PMRs would then be driven largely by differences in smoking. The strength of the correlation that we found between the two diseases supported that assumption, and although not all cases of COPD are picked up from death certificates (because of competing causes of death), it seems that the PMR from COPD did provide a meaningful proxy for smoking, making our expected numbers of deaths more reliable than would have been the case without adjustment.

We know from other research that smoking and asbestos interact in causing lung cancer, such that relative risks from the two causes in combination are more than additive.[8,9] It follows, that in a person with lung cancer who has been both a smoker and exposed to asbestos, the disease may be attributable to both causes (or put another way, avoidance of either of the exposures might have been sufficient to prevent the disease). However, with the method of statistical analysis that we employed, interactions between smoking and asbestos could be ignored. The parameter on which we focused was the difference between the number of deaths from lung cancer that actually occurred in the job group and the number that would have been expected if the job group had the smoking habits that it did, but no exposure to asbestos. That measure will have included excess deaths attributable to asbestos alone in non-smokers, and to the joint effects of smoking and asbestos as compared with smoking alone in smokers.

The variability that we found in the ratio of excess lung cancer to mesothelioma by job group may in part reflect differences by type of asbestos. Previous meta-analysis of cohort studies has suggested a lower ratio for crocidolite (0.7) than for chrysotile (6.1), amosite (4.0) and

For peer review only

mixed fibres (1.9).[6] However, intensity and timing of exposure could also be a factor, and might explain why, when we included mesotheliomas other than of the peritoneum, the mean ratio that we observed across all 22 job groups (0.28) was relatively low. Another analysis, based on national data for England and Wales during 1980-2000, suggested an intermediate ratio in the order of 0.67-1.0.[1] The disparity from our estimate may in part reflect differences in the methods used to control for confounding effects of smoking, but there may also have been changes over time in patterns of exposure to asbestos, and a reduction in the prevalence of smoking in asbestos-exposed occupations (if the joint effect of asbestos and smoking on lung cancer is more than additive, then a given exposure to asbestos will cause more lung cancers in smokers than in the same number of non-smokers). The lower ratio that we observed suggests that the current burden of lung cancer from occupational exposure to asbestos in Britain may not be so high as previously has been thought.[2]

The potential for variability in the ratio of excess lung cancer to mesothelioma should be taken into account when estimating population burdens of the disease from occupational exposure to asbestos.

Acknowledgements

We thank the staff of the Office for National Statistics, who provided us with the data files for our analysis, and Vanessa Cox, who helped with computer analysis.

Funding statement

For peer review only

This work was supported by Unit core funding from the Medical Research Council grant numbers 10.13039/5011000002 65, MRC_MC_UP_A620_1018, MRC_MC_UU_12011/5 (https://mrc.ukri.org/).

Competing interests

The authors have declared that no competing interests exist.

Authors contributions

DC designed the study, ECH and DC acquired the ONS data, AD the HSE data, and all authors developed the methodology and analysis. SD and AD carried out the analyses, ECH and DC wrote the first draft of the manuscript, and all authors revised and approved the final version.

Data sharing

The data underlying the results presented in the study can be made available to other researchers subject to agreement from the Office for National Statistics.

Data relevant to the specific occupations in this analysis are provided in the supporting information file: Supplementary Table 2.

2001-2010 occupational mortality data for England and Wales is available at https://www.ons.gov.uk/peoplepopulationandcommunity/healthandsocialcare/healthinequalities/adhocs/007958occupationalmortalityinenglandandwales2001to2010

1991-2000 occupational mortality data for England and Wales is available at https://webarchive.nationalarchives.gov.uk/20160129235354/http://www.ons.gov.uk/ons/publications/re-reference-tables.html?edition=tcm%3A77-168405

For peer review only

References

1 Darnton AJ, McElvenny DM, Hodgson JT. Estimating the number of asbestos-related lung cancer deaths in Great Britain from 1980 to 2000. *Ann Occup Hyg* 2006;50:29-38.

2 Health and Safety Executive. Asbestos-related disease statistics in Great Britain, 2019. https://www.hse.gov.uk/statistics/causdis/asbestos-related-disease.pdf Accessed February 2020.

3 Marinaccio A, Scarselli A, Binazzi A, et al. Magnitude of asbestos-related lung cancer mortality in Italy. *Br J Cancer* 2008;99:173-5.

4 Pasetto R, Terracini B, Marsili D, et al. Occupational burden of asbestos-related cancer in Argentina, Brazil, Colombia and Mexico. *Ann Global Health* 2014:80;263-68.

5 Van der Bij S, Vermeulen RCH, Portengen L, et al. Expected number of asbestos-related lung cancers in the Netherlands in the next two decades: a comparison of methods. *Occup Environ Med* 2016;73:342-49.

6 McCormack V, Peto J, Byrnes G, et al. Estimating the asbestos-related lung cancer burden from mesothelioma mortality. *Br J Cancer* 2012;106:575-84.

7 Gilham S, Rake C, Burdett G et al. Pleural mesothelioma and lung cancer risks in relation to occupational history and asbestos lung cancer burden. *Occup Environ Med* 2016;73:290-99.

8 Frost G, Darnton A, Hardin A-H. The effect of smoking on the risk of lung cancer mortality for asbestos workers in Great Britain (1971-2005). *Ann Occup Hyg* 2011;55:239-47.

9 Ngamwong Y, Tangamornsuksan W, Lohitnavy O, et al. Additive Synergism between Asbestos and Smoking in Lung Cancer Risk: A Systematic Review and Meta-Analysis. *PLoS One* 2015;10(8):e0135798

For peer review only

10 Harris EC, Palmer KT, Cox V, et al. Trends in mortality from occupational hazards among men in England and Wales during 1979-2010. *Occup Environ Med* 2016;73:385-93.

11 Health and Safety Executive. Mesothelioma mortality by occupation: Mesothelioma deaths in Great Britain for 2011-2017 and 2002-2010 by occupation. Available from: http://www.hse.gov.uk/statistics/causdis/mesothelioma/mesothelioma-mortality-by-occupation.pdf Accessed February 2020.

12 Inskip H, Coggon D, Winter P, et al. Occupational mortality of women. In: Drever F, ed. Occupational Health: Decennial Supplement. London: HMSO 1995:44-61.

13 Alderson MR. Some sources of error in British occupational mortality data. *Br J Indust Med* 1972;29:245-54.

Figure legends

Figure 1. PMRs for lung cancer and COPD in job groups with no major occupational exposure to causes of either disease: men in England and Wales aged 20-74 years, 1979-80 and 1982-2010.

Figure footnote: The areas of the circles represent the expected number of deaths from COPD in each job group over the study period. The regression line of PMR for lung cancer against PMR for COPD is from an analysis that weighted according to the expected number of deaths from COPD in each job group over the study period (see text).

Figure 2. Ratios of estimated excess deaths from lung cancer to observed deaths from cancer of pleura, 1979-80 and 1982-2010.

For peer review only

Figure footnote: a) Figures in brackets are observed numbers of deaths/corresponding PMRs for cancer of the pleura. b) Bars represent 95% confidence intervals, and the vertical line indicates the average ratio across all 22 job groups of 1.69.

Figure 3. Ratios of estimated excess deaths from lung cancer to observed deaths from cancer of pleura and mesothelioma, 2001-10.

Figure footnote: a) Figures in brackets are observed numbers of deaths/corresponding PMRs for cancer of the pleura and mesothelioma. b) Bars represent 95% confidence intervals, and the vertical line indicates the average ratio across all 22 job groups of 0.28.

For peer review only

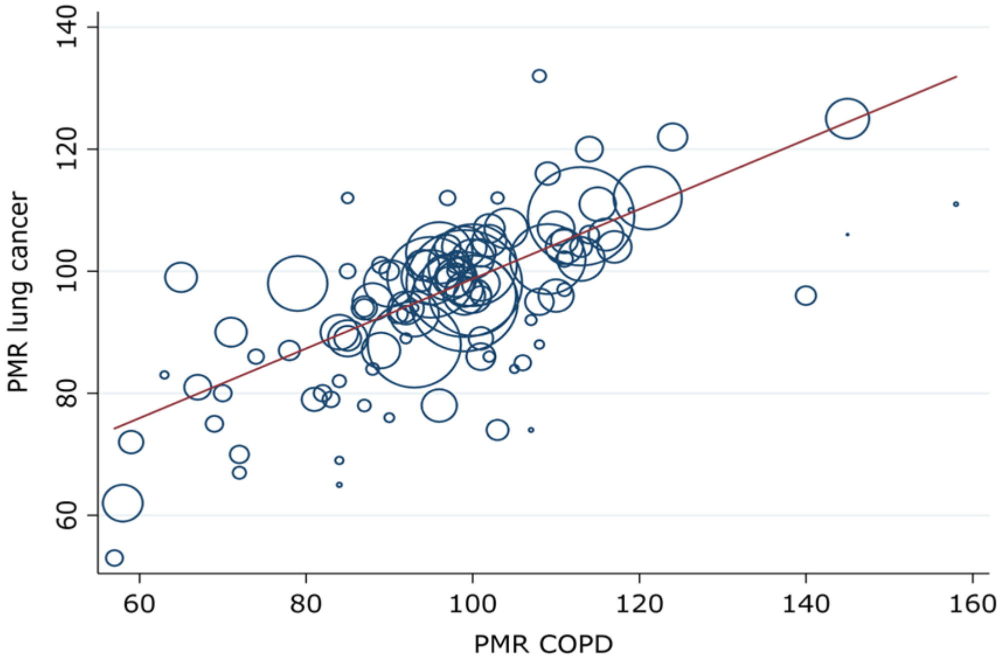

For peer review only

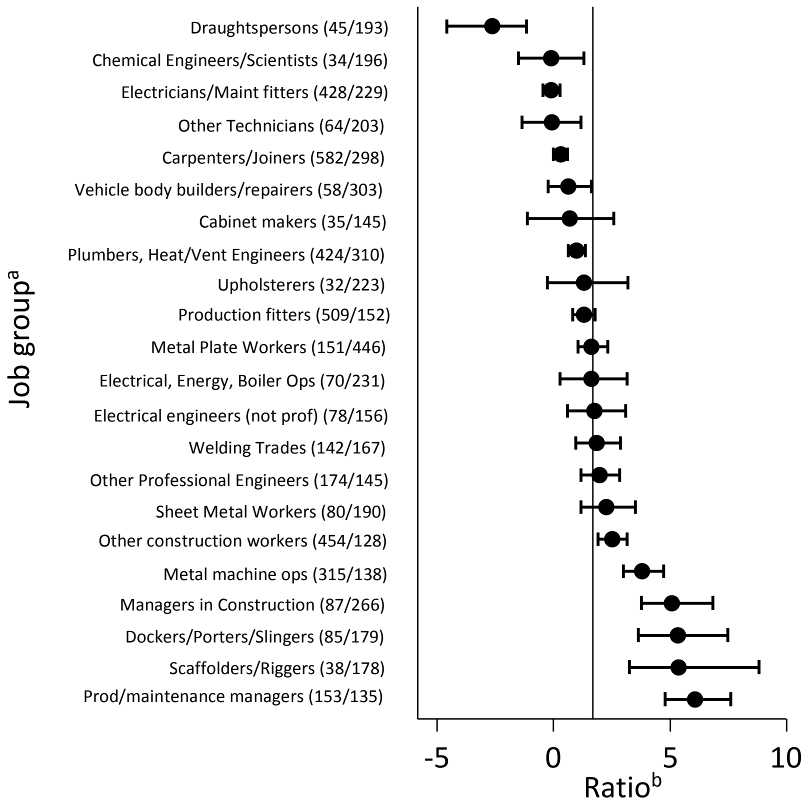

For peer review only - http://bmjopen.bmj.com/site/about/guidelines.xhtml

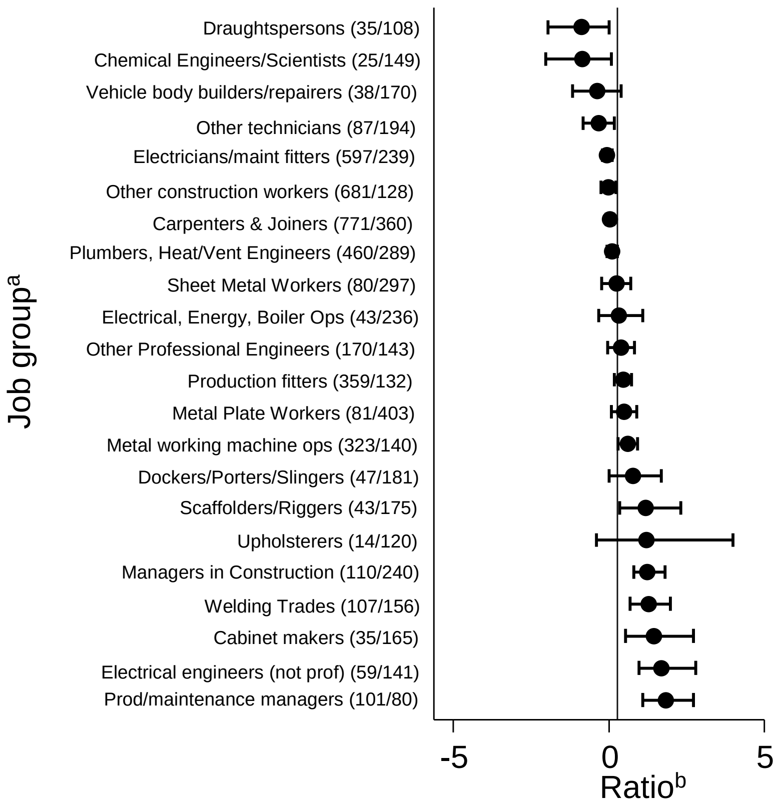
Draughtspersons (35/108)
Chemical Engineers/Scientists (25/149)
Vehicle body builders/repairers (38/170)
Other technicians (87/194)
Electricians/maint fitters (597/239)
Other construction workers (681/128)
Carpenters & Joiners (771/360)
Plumbers, Heat/Vent Engineers (460/289)
Sheet Metal Workers (80/297)
Electrical, Energy, Boiler Ops (43/236)
Other Professional Engineers (170/143)
Production fitters (359/132)
Metal Plate Workers (81/403)
Metal working machine ops (323/140)
Dockers/Porters/Slingers (47/181)
Scaffolders/Riggers (43/175)
Upholsterers (14/120)
Managers in Construction (110/240)
Welding Trades (107/156)
Cabinet makers (35/165)
Electrical engineers (not prof) (59/141)
Prod/maintenance managers (101/80)
Job group[a]
-5
0
5
Ratio[b]

review only

Supplementary Table 1: Job groups with known hazard of COPD, silica or asbestos excluded for weighting analysis

	Job group
**Excluded jobs with known hazard of COPD**	Managers in Transport, Mining and Energy Industries<br>Glass and ceramic workers combined<br>Coal miners combined<br>Moulders, Core Makers, Die Casters<br>Electroplaters combined<br>Other metal manufacturers combined
**Excluded jobs with known hazard from silica**	Chemical workers combined<br>Glass and ceramic workers combined<br>Coal miners combined<br>Moulders, Core Makers, Die Casters<br>Other metal manufacturers combined<br>Bricklayers, Masons combined<br>Mine (excluding coal) & Quarry Workers
**Excluded jobs with known hazard from asbestos**	Vocational Trainers, Social Scientists etc.<br>Chemical Engineers and Scientists<br>Other Professional Engineers<br>Draughtspersons<br>Laboratory Technicians<br>Other Technicians<br>Production and maintenance managers<br>Managers in Construction<br>Fire Service Personnel<br>Chemical workers combined<br>Upholsterers<br>Carpenters & Joiners<br>Cabinet makers combined<br>Smiths & Forge Workers<br>Metal working machine operatives combined<br>Production fitters<br>Electricians electrical maintenance fitters combined<br>Electrical engineers (not professional) combined<br>Plumbers, Heating & Ventilating Engineers & Related Trades<br>Sheet Metal Workers<br>Metal Plate Workers, Shipwrights, Riveters<br>Steel Erectors<br>Scaffolders, Riggers combined<br>Welding Trades<br>Coach and vehicle body builders and repairers combined<br>Other construction workers combined<br>Dockers goods porters and slingers combined<br>Electrical, Energy, Boiler Operatives & Attendants combined

For peer review only

Supplementary Table 2: Asbestos exposed job groups with significantly elevated PMRs for cancer of the pleura over the period 1979-2010

Job group	Deaths from all causes 1979-2010	Cancer of the pleura			Lung cancer			
		Deaths observed	Deaths expected	PMR (95%CI)	Deaths observed	Deaths expected	Deaths expected adjusted for smoking	Adjusted PMR (95%CI)
Chemical Engineers and Scientists	7,111	34	17.3	196 (136-274)	521	525.5	525.0	99 (91-108)
Other Professional Engineers	48,783	174	120.3	145 (124-168)	4,307	3,614.3	3,961.8	109 (105-112)
Draughtspersons	14,498	45	23.4	193 (141-258)	1,062	1,324.7	1,179.9	90 (85-96)
Other Technicians	17,177	64	31.6	203 (156-259)	1,499	1,480.2	1,504.3	100 (95-105)
Production and maintenance managers	63,504	153	113.3	135 (114-158)	6,418	5,827.7	5,490.1	117 (114-120)
Managers in Construction	18,079	87	32.7	266 (213-328)	2,030	1,687.6	1,589.8	128 (122-133)
Upholsterers	5,459	32	14.4	223 (152-314)	632	650.8	590.8	107 (99-116)
Carpenters & Joiners	68,780	582	195	298 (275-324)	7,566	7,987.3	7,387.8	102 (100-105)
Cabinet makers combined	9,070	35	24.1	145 (101-202)	997	1,071.8	973.0	102 (96-109)
Metal working machine operatives combined	112,777	315	228.3	138 (123-154)	13,450	13,588.2	12,258.1	110 (108-112)
Production fitters	111,536	509	334.2	152 (139-166)	13,010	13,349.8	12,347.8	105 (104-107)
Electricians electrical maintenance fitters combined	60,353	428	186.9	229 (208-252)	6,135	6,933.1	6,175.3	99 (97-102)
Electrical engineers (not professional) combined	19,392	78	49.9	156 (123-195)	1,885	2,112.4	1,748.9	108 (103-113)
Plumbers, Heating & Ventilating Engineers & Related Trades	44,862	424	136.9	310 (281-341)	5,416	5,212.5	4,999.7	108 (105-111)
Sheet Metal Workers	15,254	80	42.1	190 (151-237)	1,963	1,835.4	1,781.4	110 (105-115)
Metal Plate Workers, Shipwrights, Riveters	11,831	151	33.8	446 (378-524)	1,693	1,475.7	1,449.2	117 (111-123)
Scaffolders, Riggers combined	9,703	38	21.4	178 (126-244)	1,303	1,069.9	1,099.5	119 (112-125)
Welding Trades	30,337	142	85.0	167 (141-197)	3,897	3,555.2	3,633.3	107 (104-111)
Coach and vehicle body builders and repairers combined	6,452	58	19.1	303 (230-392)	733	739.0	696.2	105 (98-113)
Other construction workers combined	152,986	454	356	128 (116-140)	18,810	17,186.4	17,661.9	107 (105-108)
Dockers goods porters and slingers combined	26,426	85	47.6	179 (143-221)	3,667	3,332.1	3,215.1	114 (110-118)
Electrical, Energy, Boiler Operatives & Attendants combined	15,639	70	30.3	231 (180-292)	2,113	1,989.2	1,998.8	106 (101-110)

For peer review only

STROBE Statement—Checklist of items that should be included in reports of ***cross-sectional studies***

	Item No	Recommendation	Page number
**Title and abstract**	1	(*a*) Indicate the study's design with a commonly used term in the title or the abstract	Title page P2
		(*b*) Provide in the abstract an informative and balanced summary of what was done and what was found	P4-5
**Introduction**			
Background/rationale	2	Explain the scientific background and rationale for the investigation being reported	P6-7
Objectives	3	State specific objectives, including any prespecified hypotheses	P7
**Methods**			
Study design	4	Present key elements of study design early in the paper	P7-9
Setting	5	Describe the setting, locations, and relevant dates, including periods of recruitment, exposure, follow-up, and data collection	P7
Participants	6	(*a*) Give the eligibility criteria, and the sources and methods of selection of participants	P7
Variables	7	Clearly define all outcomes, exposures, predictors, potential confounders, and effect modifiers. Give diagnostic criteria, if applicable	P7-9
Data sources/ measurement	8*	For each variable of interest, give sources of data and details of methods of assessment (measurement). Describe comparability of assessment methods if there is more than one group	P7
Bias	9	Describe any efforts to address potential sources of bias	P8-9
Study size	10	Explain how the study size was arrived at	P7
Quantitative variables	11	Explain how quantitative variables were handled in the analyses. If applicable, describe which groupings were chosen and why	P8
Statistical methods	12	(*a*) Describe all statistical methods, including those used to control for confounding	P8
		(*b*) Describe any methods used to examine subgroups and interactions	P8-9
		(*c*) Explain how missing data were addressed	N/A
		(*d*) If applicable, describe analytical methods taking account of sampling strategy	N/A
		(*e*) Describe any sensitivity analyses	P9
**Results**			
Participants	13*	(a) Report numbers of individuals at each stage of study—eg numbers potentially eligible, examined for eligibility, confirmed eligible, included in the study, completing follow-up, and analysed	N/A
		(b) Give reasons for non-participation at each stage	N/A
		(c) Consider use of a flow diagram	N/A
Descriptive data	14*	(a) Give characteristics of study participants (eg demographic, clinical, social) and information on exposures and potential confounders	N/A
		(b) Indicate number of participants with missing data for each variable of interest	N/A
Outcome data	15*	Report numbers of outcome events or summary measures	Suppl Table 2 P41
Main results	16	(*a*) Give unadjusted estimates and, if applicable, confounder-adjusted estimates and their precision (eg, 95% confidence interval). Make clear which confounders were adjusted for and why they were included	Suppl Table 2 P41
		(*b*) Report category boundaries when continuous variables were categorized	N/A
		(*c*) If relevant, consider translating estimates of relative risk into absolute risk for a	N/A

For peer review only - http://bmjopen.bmj.com/site/about/guidelines.xhtml

		meaningful time period	
Other analyses	17	Report other analyses done—eg analyses of subgroups and interactions, and sensitivity analyses	P10
**Discussion**			
Key results	18	Summarise key results with reference to study objectives	P10
Limitations	19	Discuss limitations of the study, taking into account sources of potential bias or imprecision. Discuss both direction and magnitude of any potential bias	P11-13
Interpretation	20	Give a cautious overall interpretation of results considering objectives, limitations, multiplicity of analyses, results from similar studies, and other relevant evidence	P13-14
Generalisability	21	Discuss the generalisability (external validity) of the study results	P13-14
**Other information**			
Funding	22	Give the source of funding and the role of the funders for the present study and, if applicable, for the original study on which the present article is based	P14-15

*Give information separately for exposed and unexposed groups.

Note: An Explanation and Elaboration article discusses each checklist item and gives methodological background and published examples of transparent reporting. The STROBE checklist is best used in conjunction with this article (freely available on the Web sites of PLoS Medicine at http://www.plosmedicine.org/, Annals of Internal Medicine at http://www.annals.org/, and Epidemiology at http://www.epidem.com/). Information on the STROBE Initiative is available at www.strobe-statement.org.

For peer review only

For peer review only - http://bmjopen.bmj.com/site/about/guidelines.xhtml

BMJ Open

Relative burden of lung and pleural cancers from exposure to asbestos: a cross-sectional analysis of occupational mortality in England and Wales

Journal:	*BMJ Open*
Manuscript ID	bmjopen-2019-036319.R2
Article Type:	Original research
Date Submitted by the Author:	12-Mar-2020
Complete List of Authors:	Harris, Elizabeth; University of Southampton, MRC Lifecourse Epidemiology Unit; University of Southampton, MRC Versus Arthritis Centre for Musculoskeletal Health and Work<br>D'Angelo, Stefania; University of Southampton, MRC Lifecourse Epidemiology Unit; University of Southampton, MRC Versus Arthritis Centre for Musculoskeletal Health and Work<br>Darnton, Andrew; Health and Safety Executive Bootle Headquarters, Statistics and Epidemiology Unit, Science Directorate<br>Coggon, David ; University of Southampton, MRC Lifecourse Epidemiology Unit; University of Southampton, MRC Versus Arthritis Centre for Musculoskeletal Health and Work
**Primary Subject Heading**:	Epidemiology
Secondary Subject Heading:	Occupational and environmental medicine
Keywords:	EPIDEMIOLOGY, OCCUPATIONAL & INDUSTRIAL MEDICINE, Epidemiology < ONCOLOGY, Respiratory tract tumours < ONCOLOGY

SCHOLARONE™
Manuscripts

I, the Submitting Author has the right to grant and does grant on behalf of all authors of the Work (as defined in the below author licence), an exclusive licence and/or a non-exclusive licence for contributions from authors who are: i) UK Crown employees; ii) where BMJ has agreed a CC-BY licence shall apply, and/or iii) in accordance with the terms applicable for US Federal Government officers or employees acting as part of their official duties; on a worldwide, perpetual, irrevocable, royalty-free basis to BMJ Publishing Group Ltd ("BMJ") its licensees and where the relevant Journal is co-owned by BMJ to the co-owners of the Journal, to publish the Work in this journal and any other BMJ products and to exploit all rights, as set out in our licence.

The Submitting Author accepts and understands that any supply made under these terms is made by BMJ to the Submitting Author unless you are acting as an employee on behalf of your employer or a postgraduate student of an affiliated institution which is paying any applicable article publishing charge ("APC") for Open Access articles. Where the Submitting Author wishes to make the Work available on an Open Access basis (and intends to pay the relevant APC), the terms of reuse of such Open Access shall be governed by a Creative Commons licence – details of these licences and which Creative Commons licence will apply to this Work are set out in our licence referred to above.

Other than as permitted in any relevant BMJ Author's Self Archiving Policies, I confirm this Work has not been accepted for publication elsewhere, is not being considered for publication elsewhere and does not duplicate material already published. I confirm all authors consent to publication of this Work and authorise the granting of this licence.

For peer review only

Relative burden of lung and pleural cancers from exposure to asbestos: a cross-sectional analysis of occupational mortality in England and Wales

E Clare Harris[1,2]*, Stefania D'Angelo[1,2], Andrew Darnton[3], David Coggon[1,2]

[1]MRC Lifecourse Epidemiology Unit, University of Southampton, Southampton, UK

[2]MRC Versus Arthritis Centre for Musculoskeletal Health and Work, University of Southampton, Southampton, UK

[3]Statistics and Epidemiology Unit, Science Directorate, Health and Safety Executive, Bootle, UK

*Corresponding author

Elizabeth Clare Harris

MRC Lifecourse Epidemiology Unit (University of Southampton)

Southampton General Hospital

Tremona Road

Southampton

SO16 6YD

E-mail: ech@mrc.soton.ac.uk

Telephone: 02380 777624

For peer review only

Word count: 2,354

The Submitting Author has the right to grant and does grant on behalf of all authors of the Work (as defined in the below author licence), an exclusive licence and/or a non-exclusive licence for contributions from authors who are: i) UK Crown employees; ii) where BMJ has agreed a CC-BY licence shall apply, and/or iii) in accordance with the terms applicable for US Federal Government officers or employees acting as part of their official duties; on a worldwide, perpetual, irrevocable, royalty-free basis to BMJ Publishing Group Ltd ("BMJ") its licensees and where the relevant Journal is co-owned by BMJ to the co-owners of the Journal, to publish the Work in BMJ Open and any other BMJ products and to exploit all rights, as set out in our licence.

The Submitting Author accepts and understands that any supply made under these terms is made by BMJ to the Submitting Author unless you are acting as an employee on behalf of your employer or a postgraduate student of an affiliated institution which is paying any applicable article publishing charge ("APC") for Open Access articles. Where the Submitting Author wishes to make the Work available on an Open Access basis (and intends to pay the relevant APC), the terms of reuse of such Open Access shall be governed by a Creative Commons licence – details of these licences and which Creative Commons licence will apply to this Work are set out in our licence referred to above.

For peer review only

Abstract

Objectives:

To explore the extent to which asbestos-exposed jobs vary in the ratio of excess mortality from lung cancer to deaths from pleural cancer.

Design:

Using data on underlying cause of death and last full-time occupation for 3,688,916 deaths among men aged 20-74 years in England and Wales during 1979-2010, we calculated proportional mortality ratios (PMRs), standardised for age and social class, with all occupations combined as reference. For each of 22 asbestos-exposed job groups with significantly elevated PMRs for pleural cancer, we calculated excess mortality from lung cancer (observed minus expected deaths) and its ratio to number of deaths from pleural cancer. To reduce confounding effects of smoking, we adjusted expected deaths from lung cancer in each job group, according to a formula based on its PMR for chronic obstructive pulmonary disease (COPD).

Setting:

England and Wales

Participants:

3,688,916 men who died aged 20-74 years during 1979-2010

Outcome measures:

Ratios of excess mortality from lung cancer to deaths from pleural cancer by job group

Results:

Adjusted PMRs for lung cancer were elevated in all but four of the 22 asbestos-exposed job-groups, but the ratio of excess lung cancer to deaths from pleural cancer varied widely between job groups, being significantly greater than the overall ratio in six, and significantly less in seven. Analysis for 2001-2010, when (because of changes in coding) ascertainment of pleural tumours was more reliable, showed similar variation between job groups, and indicated an overall ratio of 0.28.

Conclusions:

For peer review only

Excess lung cancer in asbestos-exposed jobs is not in simple proportion to deaths from pleural cancer, and the ratio may vary importantly according to intensity of exposure to different types of asbestos and concomitant smoking habits. The current burden of lung cancer from occupational exposure to asbestos in Britain may not be so high as previously thought.

Key words: Lung cancer, pleural cancer, asbestos, mortality, occupation

For peer review only

Strengths and limitations of this study

- Use of national data covering more than 30 years gave excellent statistical precision.
- Confounding by differences in smoking habits between occupations was addressed by a novel method of adjustment based on PMRs for COPD.
- By adjusting PMRs for social class, we reduced the potential for bias because overall mortality in a job group was unusually high or low.
- There was potential for bias from misclassification of occupations and causes of death, but misclassification of lung cancer and COPD as causes of death is likely to have been non-differential with respect to occupation, and therefore to have biased PMRs for those diseases towards the null.
- There was incomplete ascertainment of pleural cancers before 2001 because deaths ascribed to mesothelioma without any specified anatomical location (most of which would have been pleural) were classed along with other cancers of unknown origin, but a separate analysis for 2001-10 that included unspecified mesotheliomas supported the main study findings.

For peer review only

Introduction

Quantifying the population burden of lung cancer from occupational exposure to asbestos is important for prioritisation of control measures, planning future healthcare provision, and assessing the impact of preventive strategies. Attributable numbers of deaths have been estimated in several countries including Great Britain,[1,2] Italy,[3] and Argentina, Brazil, Colombia and Mexico.[4] However, the task is complicated by uncertainty about the distribution of exposures across occupations, and the potential for confounding by smoking.

One approach has been to assume that impact is in proportion to the occurrence of mesothelioma. For example, when modelling future numbers of asbestos-related lung tumours in the Netherlands, Van der Bij and colleagues applied a multiplier of 1.5 to deaths from mesothelioma[5] – a factor which they derived from an earlier meta-analysis of 55 cohort studies of asbestos workers.[6] Others have suggested somewhat lower ratios of 0.55,[7] between 0.67 and 1,[1] and 1.1.[3]

One reason for variation in the ratio could be differences in smoking habits, both between countries and within a single country over time, since the combined effects of asbestos and smoking on risk of lung cancer appear to be more than additive.[8,9] In addition, the ratio of excess lung cancer to mesothelioma may vary according to intensity and duration of exposure to different types of asbestos.[6] If so, such variation could lead to differences between asbestos-exposed occupations, according to the nature of their asbestos exposure.

To explore how much the ratio of excess mortality from lung cancer to deaths from mesothelioma differs between occupations, we estimated and compared such ratios for 22 asbestos-exposed job groups, using data from a national analysis of proportionate mortality by occupation in England and Wales. As part of the analysis, we applied a novel method to adjust for potential confounding effects of smoking.

Methods

The Office for National Statistics (ONS) provided us with data on underlying cause of death and last full-time occupation for 3,688,916 deaths among men aged 20-74 years in England and Wales during 1979-2010 (excluding 1981 when records were incomplete). From these, we calculated proportional mortality ratios (PMRs), standardised for age (in five-year bands), social class (six categories) and calendar period (1979-90, 1991-2000, 2001-10), for

For peer review only

occupational categories (job groups) classified as in earlier analyses,[10] taking all occupations combined as the standard.

To address possible confounding by smoking, the prevalence of which varies by occupation, we used PMRs for chronic obstructive pulmonary disease (COPD) to adjust expected numbers of deaths from lung cancer. We first excluded job groups with excess mortality from one or more of COPD, cancer of the pleura or peritoneum, asbestosis or silicosis, which was likely to have arisen from exposures in those jobs (Supplementary Table 1). For the 106 job groups that remained (which were presumed to have no major occupational hazard of lung cancer or COPD), we confirmed that the PMR for lung cancer was linearly related to that for COPD by calculation of a Spearman correlation coefficient, and then fitted a weighted linear regression model of the form:

$$\text{(PMR for lung cancer)} = a * \text{(PMR for COPD)} + b \qquad \{1\}$$

For this purpose, the weighting was according to the expected number of deaths from COPD in each job group.

Next, we focused on 22 asbestos-exposed job groups with significantly elevated PMRs over the period 1979-2010 for cancer of the pleura (ICD9 163, ICD10 C38.4, C38.8 and C45.0, lower 95% confidence limit > 100) (Supplementary Table 2). For these job groups, we used the regression coefficients, a and b, from {1} to adjust expected numbers of deaths from lung cancer according to the PMR for COPD. Thus, the expected number of deaths was multiplied by {a * (PMR for COPD) + b}.

With this correction, we calculated the excess of lung cancer for each job group (observed – expected deaths), and its ratio to the observed number of deaths from cancer of the pleura. Confidence intervals for ratios were computed through random simulations (1000 per estimate) in which we assumed that the expected number of deaths from lung cancer was constant, while the numbers of deaths from lung cancer and cancer of the pleura each

For peer review only

followed a Poisson distribution with mean equal to the observed number of deaths from that cancer in our dataset.

During 1979-2000, when ICD 9 was used to classify causes of death, there was no separate diagnostic category for mesotheliomas with unspecified anatomical origin, and they were included in a much larger grouping of “malignant neoplasms without specification of site”. However, ICD 10, which was used during 2001-10, included unique codes for mesothelioma including C45.9 for “mesothelioma unspecified”. In a sensitivity analysis, we repeated our calculations for this period, aggregating all deaths from mesotheliomas other than of the peritoneum (C45.2, C45.7 and C45.9) with those from pleural cancer.

In addition, PMRs for deaths where mesothelioma was mentioned anywhere in the death certificate text were available for the periods 1980, 1982-2000, and 2002-2010, from national statistics published by the Health and Safety Executive (HSE).[11] In further sensitivity analyses, we related excess mortality from lung cancer by job group to excess deaths from mesothelioma in these data (adjusting the ratios to account for there being slightly fewer years of data on mesothelioma).

For peer review only

Patient and Public Involvement

This research was done without patient or public involvement.

Results

In the 106 job groups with no major hazard of COPD, silicosis or asbestos-related disease, PMRs for lung cancer correlated strongly with those for COPD (Spearman correlation coefficient = 0.78, Figure 1). The weighted regression equation was:

(PMR for lung cancer) = 0.57*(PMR for COPD) + 42.

When the coefficients from this equation were used to adjust expected numbers of lung cancer deaths in the 22 job groups with significantly high PMRs for pleural cancer (Supplementary Table 2), the PMR for lung cancer was elevated in all but four, and the overall excess of lung cancer was 1.69 times the number of deaths from pleural cancer. However, the ratio between excess deaths from lung cancer and deaths from pleural cancer varied between job groups, such that in six it was significantly greater than the overall average, and in seven significantly less (Figure 2). For completeness, Supplementary Table 3 shows this job-specific ratio stratified also by time-period (1979-1990, 1991-2000 and 2001-2010).

During 2001-2010, 3061 deaths from mesotheliomas other than of the peritoneum were recorded in the 22 asbestos-exposed job groups of interest, in addition to the 1205 classed as pleural cancer. Inclusion of these additional deaths in our calculations gave a lower overall ratio (0.28), but again indicated substantial heterogeneity between job groups (Figure 3). Moreover the job groups with the highest and lowest ratios were much the same as in the previous analysis.

Similar results were obtained in the analysis based on deaths with any mention of mesothelioma in the death certificate text. The overall ratio (in this case to excess rather than total deaths from mesothelioma) was 1.13 for the full study period, and 0.46 for 2001-2010, with similar variation in the ratios for specific job groups.

Discussion

Our analysis indicates that among occupations entailing exposure to asbestos, the ratio between excess deaths from lung cancer and deaths from pleural cancer/mesothelioma can vary substantially. This suggests that burdens of lung cancer attributable to asbestos are not

For peer review only

in simple proportion to numbers of mesotheliomas, and that the ratio may vary importantly according to the pattern of exposures within a population, and perhaps also smoking habits.

We limited our investigation to men since asbestos-related disease was much less frequent among women. Moreover, only 30% of the women who died in the early part of the study period (1979-1990) had occupations recorded on their death certificates.[12]

Our use of national data covering more than 30 years gave excellent statistical precision, but there was potential for bias from misclassification of occupations and causes of death. In the UK, death certificates document only the last full-time occupation, but for chronic diseases with long induction periods (such as lung and pleural cancer), jobs held earlier in life may be more relevant. Furthermore, occupations and causes of death are not always assigned accurately.[13] Nevertheless, we think it unlikely that such errors could account for the variation in ratios of excess lung cancer to pleural cancer that we observed.

The 22 job groups on which we focused in our main analysis were those that we could be reasonably confident were associated with an asbestos hazard. However, it was not essential that they should account for all asbestos-related cancer in the study population. Any under-ascertainment of cases attributable to work in those jobs, either because of migration to other employment or through misclassification of occupations on death certificates, would reduce both the excess mortality from lung cancer and the number of deaths from pleural cancer. However, it would not be expected to bias the ratio of those measures differentially across job groups.

For peer review only

Misclassification of lung cancer and COPD as causes of death is likely to have been non-differential with respect to occupation, and therefore to have biased PMRs for those diseases towards the null. It is reassuring, however, that after exclusion of job groups with exposure to known causes of lung cancer and/or COPD, we observed a strong correlation between PMRs for the two diseases (r = 0.78). This suggests that such misclassification was not a major problem.

A greater concern was the incomplete ascertainment of mesotheliomas before 2001 in our main dataset. This occurred because at that time, deaths ascribed to mesothelioma without any specified anatomical location (most of which would have been pleural) were classed along with other cancers of unknown origin. Data from 2001-10, when they were assigned to a specific code, indicated that they outnumbered deaths ascribed to pleural cancer more than twofold. Thus, variation in the extent of under-ascertainment by job group could have caused serious bias. However, when we restricted our analysis to 2001-10, and included mesotheliomas other than of the peritoneum with pleural cancers, there was still marked variation in their frequency relative to excess lung cancer. And importantly, the job groups with the highest and lowest ratios were much the same. Moreover, similar heterogeneity was observed in our analysis based on deaths with any mention of mesothelioma on the death certificate.

As with all analyses of proportionate mortality, there was a possibility that expected numbers of deaths from specific causes of death could be biased if overall mortality in a job group were unusually high or low. However, in stratifying our analyses by social class, we reduced the potential for large variation between job groups in total mortality, and it seems unlikely that such bias could explain differences in the ratio of excess lung cancer to pleural cancer of the magnitude that we observed.

For peer review only

A particular challenge in studying occupational mortality from lung cancer is the scope for confounding by differences in smoking habits between occupations. To address that problem, we adjusted expected deaths from lung cancer according to the PMR for COPD in the job group under consideration. In deriving the formula for the adjustment, we took care to exclude job groups with exposure to major occupational causes of either COPD or lung cancer, in the expectation that the variation between job groups in PMRs would then be driven largely by differences in smoking. The strength of the correlation that we found between the two diseases supported that assumption, and although not all cases of COPD are picked up from death certificates (because of competing causes of death), it seems that the PMR from COPD did provide a meaningful proxy for smoking, making our expected numbers of deaths more reliable than would have been the case without adjustment.

We know from other research that smoking and asbestos interact in causing lung cancer, such that relative risks from the two causes in combination are more than additive.[8,9] It follows, that in a person with lung cancer who has been both a smoker and exposed to asbestos, the disease may be attributable to both causes (or put another way, avoidance of either of the exposures might have been sufficient to prevent the disease). However, with the method of statistical analysis that we employed, interactions between smoking and asbestos could be ignored. The parameter on which we focused was the difference between the number of deaths from lung cancer that actually occurred in the job group and the number that would have been expected if the job group had the smoking habits that it did, but no exposure to asbestos. That measure will have included excess deaths attributable to asbestos alone in non-smokers, and to the joint effects of smoking and asbestos as compared with smoking alone in smokers.

For peer review only

The variability that we found in the ratio of excess lung cancer to mesothelioma by job group may in part reflect differences by type of asbestos. Previous meta-analysis of cohort studies has suggested a lower ratio for crocidolite (0.7) than for chrysotile (6.1), amosite (4.0) and mixed fibres (1.9).[6] However, intensity and timing of exposure could also be a factor, and might explain why, when we included mesotheliomas other than of the peritoneum, the mean ratio that we observed across all 22 job groups (0.28) was relatively low. Another analysis, based on national data for England and Wales during 1980-2000, suggested an intermediate ratio in the order of 0.67-1.0.[1] The disparity from our estimate may in part reflect differences in the methods used to control for confounding effects of smoking, but there may also have been changes over time in patterns of exposure to asbestos, and a reduction in the prevalence of smoking in asbestos-exposed occupations (if the joint effect of asbestos and smoking on lung cancer is more than additive, then a given exposure to asbestos will cause more lung cancers in smokers than in the same number of non-smokers). The lower ratio that we observed suggests that the current burden of lung cancer from occupational exposure to asbestos in Britain may not be so high as previously has been thought.[2]

The potential for variability in the ratio of excess lung cancer to mesothelioma should be taken into account when estimating population burdens of the disease from occupational exposure to asbestos.

Acknowledgements

We thank the staff of the Office for National Statistics, who provided us with the data files for our analysis, and Vanessa Cox, who helped with computer analysis.

For peer review only

Funding statement

This work was supported by Unit core funding from the Medical Research Council grant numbers 10.13039/5011000002 65, MRC_MC_UP_A620_1018, MRC_MC_UU_12011/5 (https://mrc.ukri.org/).

Competing interests

The authors have declared that no competing interests exist.

Authors contributions

DC designed the study, ECH and DC acquired the ONS data, AD the HSE data, and all authors developed the methodology and analysis. SD and AD carried out the analyses, ECH and DC wrote the first draft of the manuscript, and all authors revised and approved the final version.

Data sharing

The data underlying the results presented in the study can be made available to other researchers subject to agreement from the Office for National Statistics.

Data relevant to the specific occupations in this analysis are provided in the supporting information file: Supplementary Table 2.

2001-2010 occupational mortality data for England and Wales are available at https://www.ons.gov.uk/peoplepopulationandcommunity/healthandsocialcare/healthinequalities/adhocs/007958occupationalmortalityinenglandandwales2001to2010

1991-2000 occupational mortality data for England and Wales are available at https://webarchive.nationalarchives.gov.uk/20160129235354/http:/www.ons.gov.uk/ons/publications/re-reference-tables.html?edition=tcm%3A77-168405

For peer review only

References

1 Darnton AJ, McElvenny DM, Hodgson JT. Estimating the number of asbestos-related lung cancer deaths in Great Britain from 1980 to 2000. *Ann Occup Hyg* 2006;50:29-38.

2 Health and Safety Executive. Asbestos-related disease statistics in Great Britain, 2019. https://www.hse.gov.uk/statistics/causdis/asbestos-related-disease.pdf Accessed February 2020.

3 Marinaccio A, Scarselli A, Binazzi A, et al. Magnitude of asbestos-related lung cancer mortality in Italy. *Br J Cancer* 2008;99:173-5.

4 Pasetto R, Terracini B, Marsili D, et al. Occupational burden of asbestos-related cancer in Argentina, Brazil, Colombia and Mexico. *Ann Global Health* 2014:80;263-68.

5 Van der Bij S, Vermeulen RCH, Portengen L, et al. Expected number of asbestos-related lung cancers in the Netherlands in the next two decades: a comparison of methods. *Occup Environ Med* 2016;73:342-49.

6 McCormack V, Peto J, Byrnes G, et al. Estimating the asbestos-related lung cancer burden from mesothelioma mortality. *Br J Cancer* 2012;106:575-84.

7 Gilham S, Rake C, Burdett G et al. Pleural mesothelioma and lung cancer risks in relation to occupational history and asbestos lung cancer burden. *Occup Environ Med* 2016;73:290-99.

8 Frost G, Darnton A, Hardin A-H. The effect of smoking on the risk of lung cancer mortality for asbestos workers in Great Britain (1971-2005). *Ann Occup Hyg* 2011;55:239-47.

For peer review only

9 Ngamwong Y, Tangamornsuksan W, Lohitnavy O, et al. Additive Synergism between Asbestos and Smoking in Lung Cancer Risk: A Systematic Review and Meta-Analysis. *PLoS One* 2015;10(8):e0135798

10 Harris EC, Palmer KT, Cox V, et al. Trends in mortality from occupational hazards among men in England and Wales during 1979-2010. *Occup Environ Med* 2016;73:385-93.

11 Health and Safety Executive. Mesothelioma mortality by occupation: Mesothelioma deaths in Great Britain for 2011-2017 and 2002-2010 by occupation. Available from: http://www.hse.gov.uk/statistics/causdis/mesothelioma/mesothelioma-mortality-by-occupation.pdf Accessed February 2020.

12 Inskip H, Coggon D, Winter P, et al. Occupational mortality of women. In: Drever F, ed. Occupational Health: Decennial Supplement. London: HMSO 1995:44-61.

13 Alderson MR. Some sources of error in British occupational mortality data. *Br J Indust Med* 1972;29:245-54.

Figure legends

Figure 1. PMRs for lung cancer and COPD in job groups with no major occupational exposure to causes of either disease: men in England and Wales aged 20-74 years, 1979-80 and 1982-2010.

Figure footnote: The areas of the circles represent the expected number of deaths from COPD in each job group over the study period. The regression line of PMR for lung cancer against PMR for COPD is from an analysis that weighted according to the expected number of deaths from COPD in each job group over the study period (see text).

For peer review only

Figure 2. Ratios of estimated excess deaths from lung cancer to observed deaths from cancer of pleura, 1979-80 and 1982-2010.

Figure footnote: a) Figures in brackets are observed numbers of deaths/corresponding PMRs for cancer of the pleura. b) Bars represent 95% confidence intervals, and the vertical line indicates the average ratio across all 22 job groups of 1.69.

Figure 3. Ratios of estimated excess deaths from lung cancer to observed deaths from cancer of pleura and mesothelioma, 2001-10.

Figure footnote: a) Figures in brackets are observed numbers of deaths/corresponding PMRs for cancer of the pleura and mesothelioma. b) Bars represent 95% confidence intervals, and the vertical line indicates the average ratio across all 22 job groups of 0.28.

For peer review only

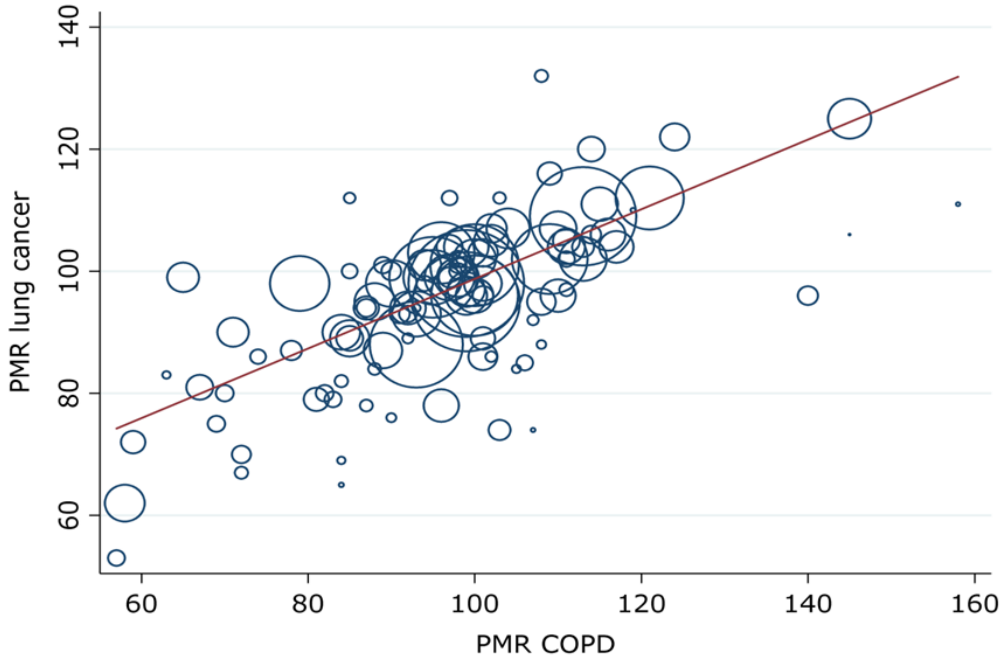

For peer review only

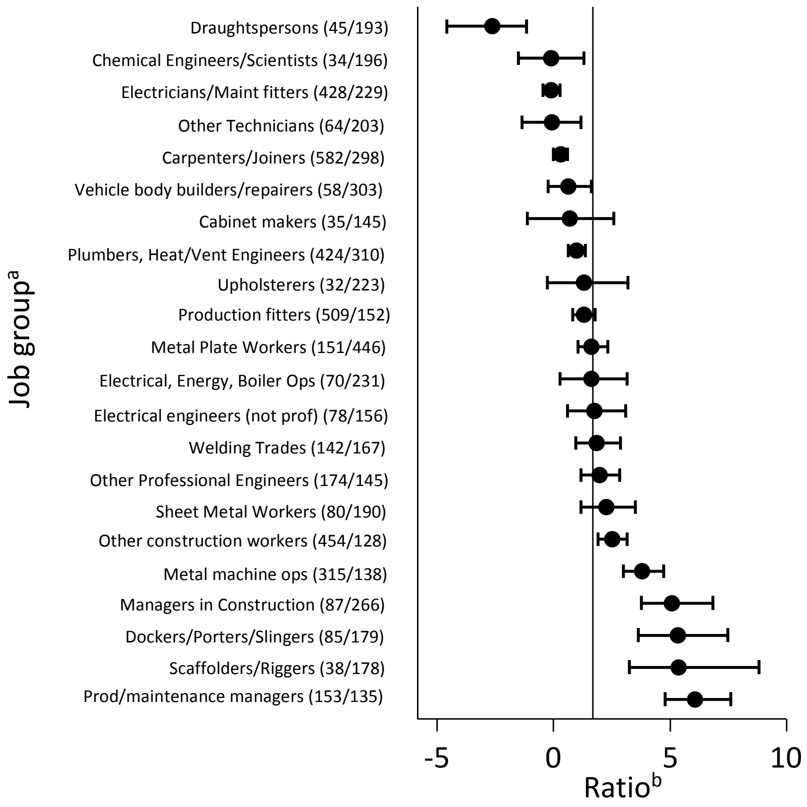
Draughtspersons (45/193)
Chemical Engineers/Scientists (34/196)
Electricians/Maint fitters (428/229)
Other Technicians (64/203)
Carpenters/Joiners (582/298)
Vehicle body builders/repairers (58/303)
Cabinet makers (35/145)
Plumbers, Heat/Vent Engineers (424/310)
Upholsterers (32/223)
Production fitters (509/152)
Metal Plate Workers (151/446)
Electrical, Energy, Boiler Ops (70/231)
Electrical engineers (not prof) (78/156)
Welding Trades (142/167)
Other Professional Engineers (174/145)
Sheet Metal Workers (80/190)
Other construction workers (454/128)
Metal machine ops (315/138)
Managers in Construction (87/266)
Dockers/Porters/Slingers (85/179)
Scaffolders/Riggers (38/178)
Prod/maintenance managers (153/135)
Job group[a]
-5
0
5
10
Ratio[b]

review only

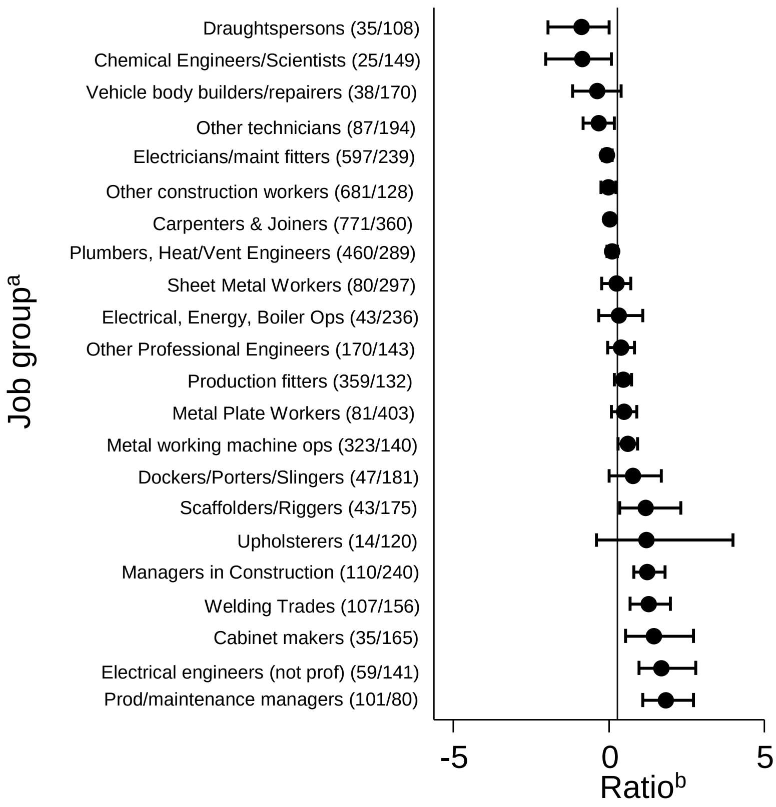
Job group[a]
Draughtspersons (35/108)
Chemical Engineers/Scientists (25/149)
Vehicle body builders/repairers (38/170)
Other technicians (87/194)
Electricians/maint fitters (597/239)
Other construction workers (681/128)
Carpenters & Joiners (771/360)
Plumbers, Heat/Vent Engineers (460/289)
Sheet Metal Workers (80/297)
Electrical, Energy, Boiler Ops (43/236)
Other Professional Engineers (170/143)
Production fitters (359/132)
Metal Plate Workers (81/403)
Metal working machine ops (323/140)
Dockers/Porters/Slingers (47/181)
Scaffolders/Riggers (43/175)
Upholsterers (14/120)
Managers in Construction (110/240)
Welding Trades (107/156)
Cabinet makers (35/165)
Electrical engineers (not prof) (59/141)
Prod/maintenance managers (101/80)
-5
0
5
Ratio[b]

review only

Supplementary Table 1: Job groups with known hazard of COPD, silica or asbestos excluded for weighting analysis

	Job group
**Excluded jobs with known hazard of COPD**	Managers in Transport, Mining and Energy Industries<br>Glass and ceramic workers combined<br>Coal miners combined<br>Moulders, Core Makers, Die Casters<br>Electroplaters combined<br>Other metal manufacturers combined
**Excluded jobs with known hazard from silica**	Chemical workers combined<br>Glass and ceramic workers combined<br>Coal miners combined<br>Moulders, Core Makers, Die Casters<br>Other metal manufacturers combined<br>Bricklayers, Masons combined<br>Mine (excluding coal) & Quarry Workers
**Excluded jobs with known hazard from asbestos**	Vocational Trainers, Social Scientists etc.<br>Chemical Engineers and Scientists<br>Other Professional Engineers<br>Draughtspersons<br>Laboratory Technicians<br>Other Technicians<br>Production and maintenance managers<br>Managers in Construction<br>Fire Service Personnel<br>Chemical workers combined<br>Upholsterers<br>Carpenters & Joiners<br>Cabinet makers combined<br>Smiths & Forge Workers<br>Metal working machine operatives combined<br>Production fitters<br>Electricians electrical maintenance fitters combined<br>Electrical engineers (not professional) combined<br>Plumbers, Heating & Ventilating Engineers & Related Trades<br>Sheet Metal Workers<br>Metal Plate Workers, Shipwrights, Riveters<br>Steel Erectors<br>Scaffolders, Riggers combined<br>Welding Trades<br>Coach and vehicle body builders and repairers combined<br>Other construction workers combined<br>Dockers goods porters and slingers combined<br>Electrical, Energy, Boiler Operatives & Attendants combined

For peer review only

Supplementary Table 2: Asbestos exposed job groups with significantly elevated PMRs for cancer of the pleura over the period 1979-2010

Job group	Deaths from all causes 1979-2010	Cancer of the pleura			Lung cancer			
		Deaths observed	Deaths expected	PMR (95%CI)	Deaths observed	Deaths expected	Deaths expected adjusted for smoking	Adjusted PMR (95%CI)
Chemical Engineers and Scientists	7,111	34	17.3	196 (136-274)	521	525.5	525.0	99 (91-108)
Other Professional Engineers	48,783	174	120.3	145 (124-168)	4,307	3,614.3	3,961.8	109 (105-112)
Draughtspersons	14,498	45	23.4	193 (141-258)	1,062	1,324.7	1,179.9	90 (85-96)
Other Technicians	17,177	64	31.6	203 (156-259)	1,499	1,480.2	1,504.3	100 (95-105)
Production and maintenance managers	63,504	153	113.3	135 (114-158)	6,418	5,827.7	5,490.1	117 (114-120)
Managers in Construction	18,079	87	32.7	266 (213-328)	2,030	1,687.6	1,589.8	128 (122-133)
Upholsterers	5,459	32	14.4	223 (152-314)	632	650.8	590.8	107 (99-116)
Carpenters & Joiners	68,780	582	195	298 (275-324)	7,566	7,987.3	7,387.8	102 (100-105)
Cabinet makers combined	9,070	35	24.1	145 (101-202)	997	1,071.8	973.0	102 (96-109)
Metal working machine operatives combined	112,777	315	228.3	138 (123-154)	13,450	13,588.2	12,258.1	110 (108-112)
Production fitters	111,536	509	334.2	152 (139-166)	13,010	13,349.8	12,347.8	105 (104-107)
Electricians electrical maintenance fitters combined	60,353	428	186.9	229 (208-252)	6,135	6,933.1	6,175.3	99 (97-102)
Electrical engineers (not professional) combined	19,392	78	49.9	156 (123-195)	1,885	2,112.4	1,748.9	108 (103-113)
Plumbers, Heating & Ventilating Engineers & Related Trades	44,862	424	136.9	310 (281-341)	5,416	5,212.5	4,999.7	108 (105-111)
Sheet Metal Workers	15,254	80	42.1	190 (151-237)	1,963	1,835.4	1,781.4	110 (105-115)
Metal Plate Workers, Shipwrights, Riveters	11,831	151	33.8	446 (378-524)	1,693	1,475.7	1,449.2	117 (111-123)
Scaffolders, Riggers combined	9,703	38	21.4	178 (126-244)	1,303	1,069.9	1,099.5	119 (112-125)
Welding Trades	30,337	142	85.0	167 (141-197)	3,897	3,555.2	3,633.3	107 (104-111)
Coach and vehicle body builders and repairers combined	6,452	58	19.1	303 (230-392)	733	739.0	696.2	105 (98-113)
Other construction workers combined	152,986	454	356	128 (116-140)	18,810	17,186.4	17,661.9	107 (105-108)
Dockers goods porters and slingers combined	26,426	85	47.6	179 (143-221)	3,667	3,332.1	3,215.1	114 (110-118)
Electrical, Energy, Boiler Operatives & Attendants combined	15,639	70	30.3	231 (180-292)	2,113	1,989.2	1,998.8	106 (101-110)

For peer review only

Supplementary Table 3: Ratio of excess deaths from lung cancer to observed deaths from cancer of the pleura, by job group and time period

	Ratio of excess deaths from lung cancer to observed deaths from cancer of the pleura (95%CI)			
**Job group**	**Whole period 1979-2010**	**Period 1 1979-1990**	**Period 2 1991-2000**	**Period 3 2001-2010**
Chemical Engineers and Scientists	-0.12 (-1.52,1.30)	1.76 (-0.43,5.41)	-0.31 (-2.41,1.81)	-3.04 (-11.57,0.27)
Other Professional Engineers	1.98 (1.18,2.84)	3.57 (1.97,5.69)	1.11 (0.10,2.22)	1.46 (-0.13,3.29)
Draughtspersons	-2.62 (-4.58,-1.16)	-4.14 (-9.12,-1.62)	-0.64 (-3.40,1.68)	-3.05 (-8.36,0.06)
Other Technicians	-0.08 (-1.34,1.18)	2.32 (-3.04,4.68)	0.01 (-1.23,1.24)	-1.28 (-3.71,0.74)
Production and maintenance managers	6.06 (4.79,7.6)	6.76 (4.68,9.77)	5.28 (3.38,8.14)	5.31 (3.06,8.82)
Managers in Construction	5.06 (3.76,6.82)	5.79 (3.55,9.67)	4.62 (2.59,8.43)	4.70 (2.89,8.01)
Upholsterers	1.29 (-0.28,3.17)	1.84 (-0.44,5.17)	-0.62 (-3.47,2.31)	2.83 (-0.86,12.12)
Carpenters & Joiners	0.31 (0,0.60)	0.98 (0.29,1.72)	-0.14 (-0.63,0.32)	0.16 (-0.27,0.58)
Cabinet makers combined	0.69 (-1.14,2.59)	-3.92 (-15.09,1.43)	0.04 (-2.42,2.63)	4.21 (1.50,10.07)
Metal working machine operatives combined	3.78 (2.99,4.73)	4.53 (3.06,6.48)	3.93 (2.68,5.53)	2.32 (1.13,3.87)
Production fitters	1.30 (0.83,1.78)	1.48 (0.62,2.41)	0.77 (0.14,1.37)	1.58 (0.62,2.61)
Electricians electrical maintenance fitters combined	-0.09 (-0.47,0.26)	-0.16 (-0.89,0.56)	0.23 (-0.48,0.92)	-0.17 (-0.69,0.32)
Electrical engineers (not professional) combined	1.74 (0.60,3.10)	0.57 (-1.27,2.69)	0.76 (-1.06,2.72)	5.53 (2.95,11.86)
Plumbers, Heating & Ventilating Engineers & Related Trades	0.98 (0.63,1.35)	2.15 (1.41,3.00)	0.53 (-0.01,1.07)	0.37 (-0.21,0.96)
Sheet Metal Workers	2.27 (1.16,3.52)	3.64 (0.94,7.57)	2.58 (0.85,5.40)	0.69 (-0.61,2.30)
Metal Plate Workers, Shipwrights, Riveters	1.61 (1.03,2.31)	1.96 (1.11,2.99)	1.27 (0.25,2.60)	1.17 (0.23,2.40)
Scaffolders, Riggers combined	5.36 (3.24,8.80)	2.83 (-0.65,9.13)	8.55 (4.75,17.67)	4.25 (1.09,10.43)
Welding Trades	1.86 (0.95,2.85)	1.26 (-0.20,2.84)	1.14 (-0.29,2.81)	3.71 (1.95,6.59)
Coach and vehicle body builders and repairers combined	0.63 (-0.25,1.62)	1.41 (0.03,3.28)	0.64 (-0.93,2.46)	-1.08 (-4.00,1.21)
Other construction workers combined	2.53 (1.91,3.16)	4.72 (3.43,6.15)	2.87 (1.90,4.03)	-0.02 (-1.09,1.04)
Dockers goods porters and slingers combined	5.32 (3.62,7.48)	7.06 (4.22,11.43)	3.79 (1.93,6.61)	4.13 (0.03,11.73)
Electrical, Energy, Boiler Operatives & Attendants combined	1.63 (0.29,3.16)	2.71 (0.75,5.29)	0.11 (-2.16,2.72)	1.30 (-1.37,5.04)

For peer review only

STROBE Statement—Checklist of items that should be included in reports of ***cross-sectional studies***

	Item No	Recommendation	Page number
**Title and abstract**	1	(*a*) Indicate the study's design with a commonly used term in the title or the abstract	Title page P2
		(*b*) Provide in the abstract an informative and balanced summary of what was done and what was found	P4-5
**Introduction**			
Background/rationale	2	Explain the scientific background and rationale for the investigation being reported	P6-7
Objectives	3	State specific objectives, including any prespecified hypotheses	P7
**Methods**			
Study design	4	Present key elements of study design early in the paper	P7-9
Setting	5	Describe the setting, locations, and relevant dates, including periods of recruitment, exposure, follow-up, and data collection	P7
Participants	6	(*a*) Give the eligibility criteria, and the sources and methods of selection of participants	P7
Variables	7	Clearly define all outcomes, exposures, predictors, potential confounders, and effect modifiers. Give diagnostic criteria, if applicable	P7-9
Data sources/ measurement	8*	For each variable of interest, give sources of data and details of methods of assessment (measurement). Describe comparability of assessment methods if there is more than one group	P7
Bias	9	Describe any efforts to address potential sources of bias	P8-9
Study size	10	Explain how the study size was arrived at	P7
Quantitative variables	11	Explain how quantitative variables were handled in the analyses. If applicable, describe which groupings were chosen and why	P8
Statistical methods	12	(*a*) Describe all statistical methods, including those used to control for confounding	P8
		(*b*) Describe any methods used to examine subgroups and interactions	P8-9
		(*c*) Explain how missing data were addressed	N/A
		(*d*) If applicable, describe analytical methods taking account of sampling strategy	N/A
		(*e*) Describe any sensitivity analyses	P9
**Results**			
Participants	13*	(a) Report numbers of individuals at each stage of study—eg numbers potentially eligible, examined for eligibility, confirmed eligible, included in the study, completing follow-up, and analysed	N/A
		(b) Give reasons for non-participation at each stage	N/A
		(c) Consider use of a flow diagram	N/A
Descriptive data	14*	(a) Give characteristics of study participants (eg demographic, clinical, social) and information on exposures and potential confounders	N/A
		(b) Indicate number of participants with missing data for each variable of interest	N/A
Outcome data	15*	Report numbers of outcome events or summary measures	Suppl Table 2 P41
Main results	16	(*a*) Give unadjusted estimates and, if applicable, confounder-adjusted estimates and their precision (eg, 95% confidence interval). Make clear which confounders were adjusted for and why they were included	Suppl Table 2 P41
		(*b*) Report category boundaries when continuous variables were categorized	N/A
		(*c*) If relevant, consider translating estimates of relative risk into absolute risk for a	N/A

For peer review only

		meaningful time period	
Other analyses	17	Report other analyses done—eg analyses of subgroups and interactions, and sensitivity analyses	P10
**Discussion**			
Key results	18	Summarise key results with reference to study objectives	P10
Limitations	19	Discuss limitations of the study, taking into account sources of potential bias or imprecision. Discuss both direction and magnitude of any potential bias	P11-13
Interpretation	20	Give a cautious overall interpretation of results considering objectives, limitations, multiplicity of analyses, results from similar studies, and other relevant evidence	P13-14
Generalisability	21	Discuss the generalisability (external validity) of the study results	P13-14
**Other information**			
Funding	22	Give the source of funding and the role of the funders for the present study and, if applicable, for the original study on which the present article is based	P14-15

*Give information separately for exposed and unexposed groups.

Note: An Explanation and Elaboration article discusses each checklist item and gives methodological background and published examples of transparent reporting. The STROBE checklist is best used in conjunction with this article (freely available on the Web sites of PLoS Medicine at http://www.plosmedicine.org/, Annals of Internal Medicine at http://www.annals.org/, and Epidemiology at http://www.epidem.com/). Information on the STROBE Initiative is available at www.strobe-statement.org.

For peer review only
